# Vibrational Spectroscopic Investigation of Blood Plasma and Serum by Drop Coating Deposition for Clinical Application

**DOI:** 10.3390/ijms22042191

**Published:** 2021-02-22

**Authors:** Jing Huang, Nairveen Ali, Elsie Quansah, Shuxia Guo, Michel Noutsias, Tobias Meyer-Zedler, Thomas Bocklitz, Jürgen Popp, Ute Neugebauer, Anuradha Ramoji

**Affiliations:** 1 Institute of Physical Chemistry and Abbe Center of Photonics, Friedrich-Schiller-University, Helmholtzweg 4, D-07743 Jena, Germany; jing_huang@fudan.edu.cn (J.H.); nairveen.ali@uni-jena.de (N.A.); elsie.quansah@uni-jena.de (E.Q.); shuxia_guo@seu.edu.cn (S.G.); tobias.meyer@leibniz-ipht.de (T.M.-Z.); thomas.bocklitz@uni-jena.de (T.B.); juergen.popp@uni-jena.de (J.P.); ute.neugebauer@med.uni-jena.de (U.N.); 2 Leibniz Institute of Photonic Technology, Member of Leibniz Health Technologies, Albert-Einstein-Straße 9, D-07745 Jena, Germany; 3 Department of Cardiology Internal Medicine, Jena University Hospital, Am Klinikum 1, D-07747 Jena, Germany; michel.noutsias@uk-halle.de; 4 Mid-German Heart Center, Department of Internal Medicine III (KIM-III), Division of Cardiology, Angiology and Intensive Medical Care, University Hospital Halle, Martin-Luther-University Halle-Wittenberg, Ernst-Grube-Strasse 40, D-06120 Halle (Saale), Germany; 5 Center for Sepsis Control and Care (CSCC), Jena University Hospital, Am Klinikum 1, D-07747 Jena, Germany; 6 InfectoGnostics Research Campus Jena, Centre of Applied Research, Philosophenweg 7, D-07743 Jena, Germany

**Keywords:** plasma, serum, coffee-ring effect, cardiac patients, vibrational spectroscopy, fluorescence lifetime

## Abstract

In recent decades, vibrational spectroscopic methods such as Raman and FT-IR spectroscopy are widely applied to investigate plasma and serum samples. These methods are combined with drop coating deposition techniques to pre-concentrate the biomolecules in the dried droplet to improve the detected vibrational signal. However, most often encountered challenge is the inhomogeneous redistribution of biomolecules due to the coffee-ring effect. In this study, the variation in biomolecule distribution within the dried-sample droplet has been investigated using Raman and FT-IR spectroscopy and fluorescence lifetime imaging method. The plasma-sample from healthy donors were investigated to show the spectral differences between the inner and outer-ring region of the dried-sample droplet. Further, the preferred location of deposition of the most abundant protein albumin in the blood during the drying process of the plasma has been illustrated by using deuterated albumin. Subsequently, two patients with different cardiac-related diseases were investigated exemplarily to illustrate the variation in the pattern of plasma and serum biomolecule distribution during the drying process and its impact on patient-stratification. The study shows that a uniform sampling position of the droplet, both at the inner and the outer ring, is necessary for thorough clinical characterization of the patient’s plasma and serum sample using vibrational spectroscopy.

## 1. Introduction

Deposition of a liquid sample such as a drop of blood plasma or serum on a solid substrate and let to dry, results in a ring shape at the edge of the dried droplet due to the well-known phenomena ‘coffee-ring effect’ [1]. The formation of coffee-ring was studied in depth by Deegan and his colleagues in 1997 [2]. Three processes mainly contribute to the formation of the coffee-ring effect: Self-pinning of the liquid-substrate contact line [3], higher evaporation rate at the contact line [3], and effects of Marangoni flow [4]. During the drying process, there is a higher evaporation rate at the pinned liquid–substrate contact line, which results in the transportation of suspended molecules from the interior towards the edge of the droplet [2,3,5]. As the liquid evaporates, the temperature gradient along the surface of the drying droplet induces thermal Marangoni flows, which carries the particles near the edge inward toward the top of the droplet and then plunges them down near the center [4,6]. After drying, the droplet will have an inhomogeneous redistribution with most molecules concentrated at the edge; this redistribution is the co-result of coffee-ring effect and suppression of thermal Marangoni effect by counteracting the outward capillary flow [4].

Besides the aforementioned effects, cracking patterns can be observed on the air-dried sample droplet. The reason for the cracks is that a liquid film is formed on the top layer of the droplet, usually during the drying process. The mismatch between the shrinkage of the film and the substrate constraint in the transverse direction gives rise to transverse tensile stress in the drying drop. Once the tensile stress exceeds the strength of the surface on the film, cracks will show up to release the stress [7,8,9,10]. The cracks may not form immediately in blood plasma and blood serum during the drying process because the soft suspended biomolecules in the plasma and serum samples can withstand more stress and reduce the evaporation rate [7,10].

As described above, when depositing plasma or serum on a CaF_2_ slide, most protein molecules will be driven toward the contact line and are pre-concentrated at the ring, while highly soluble inorganic electrolytes and some proteins are located at the center of the droplet [10]. For the coffee-ring effect, crystal patterns are usually formed in the center of the dried droplet besides the redistribution of proteins. However, the origin for the crystal patterns is still under debate [10]. Chen and Mohamed proposed that protein macromolecules and inorganic salts made up the crystal-like patterns in the center [11], while Yakhno proposes that the crystal-like patterns were only crystallized salts on top of the gelled matrix which was formed by protein macromolecules [12,13].

Although it is still unclear how the patterns form within the droplet during the drying process, the drop coating deposition method has been used for liquid sample investigation for medical diagnosis since the 1950s [14]. The traditional droplet clinical research is focused on obtaining morphological information from the dried droplet for biomedical applications, such as crystal aggregates, crystal shape and size, and crack patterns [14]. Advanced analytical methods, including Raman spectroscopy and FT-IR spectroscopy, are emerging and capable of providing biochemical information that cannot be extracted just by analysis of the droplet morphology. When combining vibrational spectroscopy with the droplet coating method for clinical application, one important issue is to find out appropriate measurement strategies representing the whole sample area. However, it needs to be faster and needs to produce data with a high signal-to-noise ratio.

Blood plasma and blood serum are complex specimens that contain information reflecting one’s inner physiological state and have been applied widely to discover disease-related biomarkers [15,16,17,18,19,20,21,22,23]. By investigating the dried blood plasma or serum patterns, it is possible to evaluate one’s health status [10,14]. However, the morphological pattern of dried sample is easily influenced by the drying conditions, the Rhesus (Rh) factor of the blood, individual diet, and the substrate material. Further, studying the morphological pattern is a subjective method [10]. To objectively interpret the morphological pattern and, ideally, to combine with the underlying chemical information will provide complete diagnostic information. Hence, we propose vibrational spectroscopic methods including Raman spectroscopy and FT-IR spectroscopy for analysis of air-dried plasma and serum samples prepared by drop coating deposition technique at room temperature ~20 °C.

Vibrational spectroscopy extracts biochemical information from the samples in a non-invasive and rapid way. Drop coating deposition Raman spectroscopy (DCDR) was first introduced by Dongmao Zhang and his colleagues in 2003 [24]. They were able to improve the detection limit by more than 1000 times compared to conventional Raman spectroscopy. Ever since, DCDR spectroscopy with blood plasma and blood serum have attracted higher interest for biomedical applications, such as differentiation of SIRS and sepsis [25], detection of glycated hemoglobin in diabetic cases [26], and diagnostics of ovarian cancer [27]. Also, the drop coating blood plasma and serum investigation with FT-IR has been widely applied for biomedicine applications, namely, diagnosis of ovarian cancer [27,28], discrimination of brain tumor severity [29], and for the liver cancer diagnostics [30]. There are many advantages to prepare body fluid samples via drop coating deposition approach: Firstly, the interference of solvents can be minimized or avoided to a certain extent, especially for FT-IR spectroscopy where water absorbs strongly; besides, the fluorescence background is reduced due to fluorescent impurities [31]; moreover, the proteins in the sample are pre-concentrated and segregated; last but not least, the required sample volume is smaller, and a few microliters would be sufficient. Nevertheless, many challenges are present and need to be solved. One challenge is to reduce the droplet size, to enable spectra collected from the whole droplet. This can be approached by robotic methods via standard laboratory techniques. Such a robotic system is costly and suitable for large-scale screening, further, interaction between sample and the micro-sampling needle material of the robotic system poses additional problem. Another challenge is to choose an appropriate measurement schematic to better represent the whole droplet to obtain a full spectrum of the chemical composition of a given sample and is applicable irrespective of sample type, variety, and heterogeneity, independent of proposed diagnostic applications, and is less time-consuming.

The challenges can be overcome by understanding the distribution of blood proteins in the dried droplet and to find out an optimum measurement schematic for different biomedical applications [10,32,33]. In this work, we study the biochemical variations present within the droplet systematically using Raman and FT-IR spectroscopy. Healthy donors were recruited to investigate the spectral difference between inner (central zone) and outer-ring regions of the droplet, and to confirm the distribution of abundant blood protein exemplarily, albumin was investigated in the dried droplet. Subsequently, two different cardiac-related diseases were investigated, these include two case studies: One patient suffering from congestive heart failure and the second case study is of an ischemic cardiomyopathy patient; in both cases, plasma and serum samples have been studied. Further, the vibrational spectroscopy findings were supported by applying the fluorescence lifetime imaging (FLIM) technique to gain insight into the different biomolecule distribution between the inner and the outer-ring region of the dried droplet.

## 2. Results and Discussions

### 2.1. Microscopic Morphology and Measurement Schematic of Dried Sample Drop

The morphology usually observed for a dried plasma droplet is shown in Figure S2A,B, ESI with two different macroscopically prominent visual patterns, i.e., inhomogeneous crystal structures in the inner region and homogeneous distribution in the outer-ring region. One can visualize the spike-shaped crystalline structure in the inner region, which is surrounded by the homogenous outer-ring region. The inner and the outer-ring regions are referred to by inner and outer-ring region in this manuscript, respectively. To determine the region of interest (ROI) within the droplet to detect disease-relevant molecular markers, systematic vibrational spectroscopic analysis of the inner and the outer-ring region of plasma and serum droplets has been presented in the following sections.

### 2.2. Vibrational Spectra Comparison between the Inner and Outer-Ring Region of Dried Sample Drop

**Figure 1:**
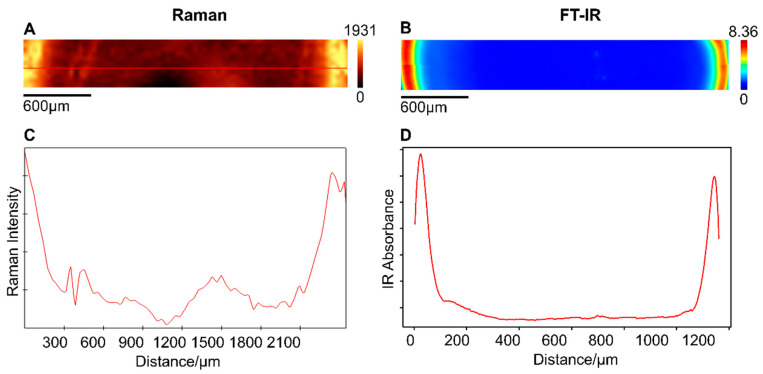
False color (**A**) Raman image and (**B**) FT-IR image of the dried plasma droplet showing intensity distribution of amide I vibrations around 1660 cm^−1^ along with (**C**) Raman and (**D**) FT-IR intensity profile from the transverse axis.

The dried sample droplet has varying morphology formed during the drying process, and the observed morphology is consistent with the literature [10,13,34]. The drying process of a colloidal solution, e.g., plasma or serum, involves complexity such as surface tension, vapor removal, or line pinning of the droplet border, which control the deposition of molecules in a coupled manner during the drying processes [35,36]. To investigate the biochemical variance between the inner and outer-ring regions of a dried plasma droplet, vibrational spectra (Raman and FT-IR) were collected from these regions (Figure S2A, ESI). In the unprocessed raw Raman and FT-IR spectra, higher intensities are observed for the spectra collected from the outer-ring region than in the inner region, for both plasma and serum samples (Figure S2C–F, ESI). This is indicative of the differences in the amount of the biomolecules between inner and outer-ring regions. Further, this difference in the spectral intensity between inner and outer-ring regions has been illustrated by recording Raman and FT-IR image from a transverse section along the diameter of the dried plasma droplet (Figure 1). An intensity profile of amide I vibration was generated based on the integrated intensity of protein vibration, with an amide I band appearing at 1660 cm^−1^ for both Raman and FT-IR raw spectral data. As can be visualized from Figure 1, the highest signal contribution comes from the outer-ring region of the droplet. Similar observations are confirmed for the serum samples as shown in Figures S2E,F and S3, ESI.

The spectral contributions are mainly from proteins, with prominent bands around amide I (between 1600 and 1700 cm^−1^), amide II (between 1500 and 1600 cm^−1^), and amide III (between 1200 and 1300 cm^−1^) bands in both Raman and FT-IR spectra (Figure S2C,D, ESI). As shown in Figure 1, for the amide I vibrations, most of the proteins aggregate in the outer-ring region, and this can be mainly attributed to the coffee-ring effect. During the drying process, the contact line, a region where the sample liquid meets the substrate [10,13], has a higher evaporation rate. Due to the outward capillary flow during the evaporation, the proteins from the inner region are carried towards the outer-ring region and deposited [33]. Therefore, most of the proteins will pre-concentrate in the outer-ring region [2,10]. However, in the inner region of the droplet, a weak vibrational signature of proteins is still observed (Figure S2C,D, ESI). The accumulation of the proteins in the inner region can be attributed to the Marangoni effect. The Marangoni effect states that, during the drying process, simultaneous suppression of the coffee-ring effect occurs. As a result of Marangoni flow, some of the biomolecules are carried toward the inner region of the droplet [4]. The distribution of biomolecules during the drying process of the sample having different particle size and charge are influenced by the coffee-ring effect [37]. This uneven distribution of the biomolecules during vibrational spectroscopic investigations of dried sample correlates directly to the spectral region of interest, which has a diagnostic impact [32,33].

### 2.3. Distribution of Abundant Blood Protein in the Dried Droplet

To better understand the distribution of biomolecules in the inner and outer-ring regions of the dried droplet, the vibrational spectra of plasma and serum were firstly preprocessed to make the spectra comparable. The spectral profile of the inner and outer-ring regions of the plasma and serum droplet have similar spectral profile and for comparison, Raman and FT-IR spectra of the albumin has been shown in Figure S4, ESI. The major contributing biomolecules are proteins, especially albumin as it forms 55% of the total blood protein composition and often plays a role as the carrier protein [38] in the blood. Hence, the vibrational spectra of the plasma and serum samples show dominating contribution coming from albumin [39]. From the spectral profiles, it can be observed that albumin localizes both in the inner and outer-ring regions of the dried droplet.

However, most of the proteins present in the plasma and serum share similar Raman spectral profile, which makes it difficult to estimate the relative abundance of albumin in the inner and outer-ring regions. With albumin being one of the markers for detection of kidney and liver diseases [40,41], it is beneficial to know the region of interest (ROI) in the sample droplet to capture and detect the albumin via vibrational spectroscopy technique.

**Figure 2:**
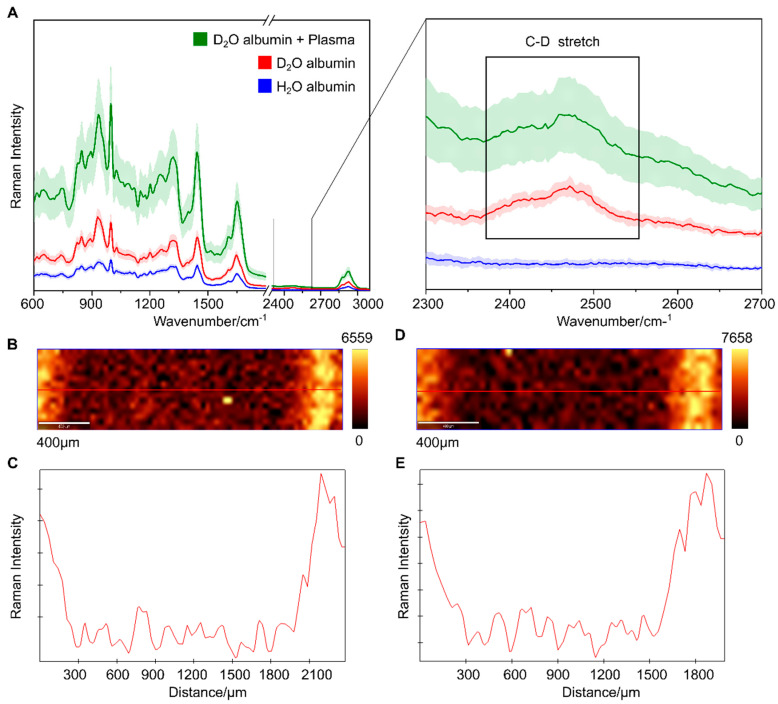
(**A**) Raman spectra comparison of deuterated (D_2_O-albumin), non-deuterated albumin (H_2_O albumin), and deuterated albumin mixed with plasma of a healthy donor in the ratio 1:2 (D_2_O albumin + Plasma). Inset shows enlarged Raman spectral region around 2300 cm^−1^ to 2700 cm^−1^. False color Raman images showing intensity distribution of C-D stretching vibration around 2470 cm^−1^ of the dried (**B**) D_2_O-albumin and (**D**) D_2_O-albumin + Plasma along with the intensity profile from the transverse axis of the dried droplet of (**C**) D_2_O-albumin and (**E**) D_2_O-albumin + Plasma.

To investigate the preferred location of albumin during the drying process, albumin was dissolved in heavy water (D_2_O) for one week. The deuterated albumin C-D stretching vibration is shifted and appears at lower wavenumber compared to the C-H stretching vibrations allowing selectively detection of the albumin location. Firstly, to evidence the hydrogen–deuterium exchange, Raman spectra of albumin dissolved in D_2_O and H_2_O have been compared in Figure 2A. In the Raman spectra of D_2_O albumin, the Raman band of C-D stretch is observed at 2470 cm^−1^ (inset for Figure 2A). However, the Raman band of C-H is also observed in the Raman spectra of D_2_O albumin indicating partial hydrogen-deuterium exchange. To visualize the distribution of albumin in the dried sample droplet, Raman images were collected from the transverse region along the diameter of the dried droplet.

An integrated intensity map of the C-D Raman band occurring at 2470 cm^−1^ was generated as shown in Figure 2B along with an intensity line profile for the horizontal axis (Figure 2C). As can be visualized from the Raman image (Figure 2B), deuterated albumin mostly localizes at the outer-ring region. Further, deuterated albumin was mixed with plasma and serum samples, to assess the effect of other plasma biomolecules on the albumin, and Raman imaging was performed similarly from the transverse region as done for the pure albumin. Similar results as described above were observed for both plasma sample mixed with D_2_O albumin (Figure 2D,E) and serum sample mixed with D_2_O albumin (Figure S5, ESI).

The higher intensity of C-D Raman band at the outer-ring indicate that deuterated-albumin mainly locates at the outer-ring region of the dried droplet in agreement with the literature describing the distribution of protein at the periphery during the drying process [33,42]. It is possible that some fraction of non-deuterated albumin orients towards the inner region during the sample drying process. However, the reason for using deuterated-albumin was mainly to show that during the drying process albumin localization gets influenced by the physical (structure e.g., β-sheet) and chemical (such albumin-bound proteins) properties of the albumin present in the sample [42].

### 2.4. PCA Analysis of Spectra between Inner and Outer-Ring Regions of Dried Sample Drop

**Figure 3:**
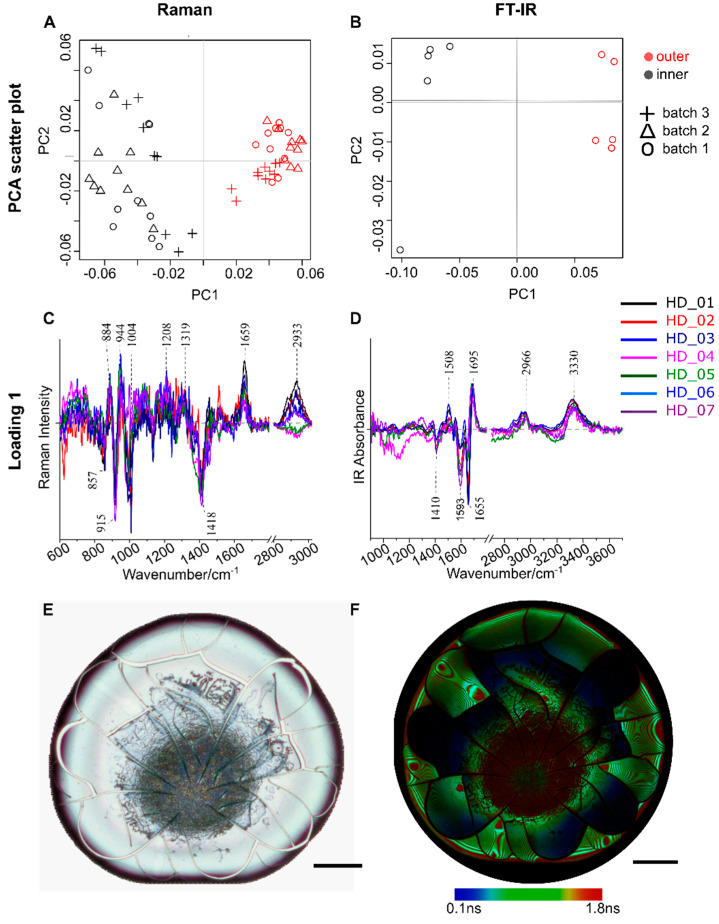
Principal component analysis (PCA) analysis of (**A**) Raman spectra and (**B**) FT-IR spectra collected from inner and outer-ring regions of the dried plasma droplet from a healthy donor (HD). (*n* = 1). PCA analysis was performed separately for *n* = 7 healthy donors; for the sake of space the PCA score plot has been shown for only one healthy donor, whereas the loadings coefficient PC1 obtained for (**C**) Raman and (**D**) FT-IR spectral data for different healthy donors (*n* = 7) overlayed on top of each other has been shown for comparison. (**E**) White light image of a healthy donor plasma (*n* = 1) shown along with the (**F**) fluorescence lifetime image to illustrate molecules having different lifetimes in the inner and outer-ring regions. Scalebar is 200 µm.

To extract vibrational spectroscopic signatures of the biomolecules between inner and outer-ring regions, PCA analysis for spectra collected from inner and outer-ring regions are performed separately for individual donors. Exemplarily, the PCA scatter plot has been presented in Figure 3A (Raman) and 3B (FT-IR) for one donor. The PCA scatter plot shows that the spectra obtained from the inner and outer-ring regions can be distinctly separated into two clusters, indicating difference in the biochemical compositions between these two regions. To better understand the biochemical differences between inner and outer-ring regions, the corresponding loadings coefficient for the principal component (PC) separating the two groups; in this case, PC1 is shown in Figure 3C (Raman) and Figure 3D (FT-IR) for all the healthy donors (*n* = 7).

The PC1 loadings show a difference in the vibrational spectral profile (Figure 3C,D) of the sample from inner and outer-ring regions. The observed Raman bands mainly have contributions from proteins e.g., Tyrosine (857 cm^−1^, 1208 cm^−1^), Proline (857 cm^−1^), Phenylalanine (1004 cm^−1^), and amide I (1659 cm^−1^). The contributions of proteins can also be confirmed in the FT-IR loading (Figure 3D) differentiating the inner and outer-ring regions, e.g., protein vibrations occurring at amide II (1508 cm^−1^), amide I with different folding (α-helix:1655 cm^−1^, β-sheet:1695 cm^−1^), and C-H stretching (2966 cm^−1^). In the case of Raman loadings (Figure 3C), the C-H stretching (2933 cm^−1^) has an opposite trend for two donors. These opposite trends near C-H stretching vibrations (2933 cm^−1^) arising due to long-chain lipids and fatty acids for different individuals (Figure 3C) might be attributed to heterogeneity among donors especially dietary lipids’ variances in plasma or serum. Since before blood collection from the healthy individuals the diet was not controlled, this is the probable cause for the variation in the intensity of lipid Raman band observed at 2933 cm^−1^. Further, it is also known that the lipids/fatty acids in the blood are very susceptible to the dietary consumption of the lipids/fatty acids intakes whereas blood proteins are quite constant regarding the dietary protein [43], thus lipid profile of plasma or serum can differ among individuals. Similar results for serum samples are depicted in Figure S6, ESI.

Further, the fluorescence lifetime imaging (FLIM) method was employed to visualize the differences in the biomolecular composition of the inner and outer-ring region of dried plasma samples. The FLIM image was acquired from a healthy donor plasma samples, and the white light image of the dried plasma sample along with the FLIM image are displayed in Figure 3E,F, respectively. From the FLIM image (Figure 3F) one can visualize that the lifetime of the contributing molecules from the inner and the outer-ring regions are different. Two distinct lifetimes “τ1 and τ2” with the values 0.18 ns and 1.86 ns were observed. The FLIM images were fitted using a 2-Exponential Reconvolution method to give two lifetimes τ1 and τ2 with values of 0.18 ns and 1.86 ns, respectively. It was observed from the FLIM image of the healthy donor that there were lifetime changes in the plasma sample depicted by the color-coded values in Figure 3, but the molecules contributing to these changes were still unclear. However, regions with different lifetime indicate different molecular composition.

The biochemical difference observed in the inner region and the outer-ring region of the dried plasma and serum samples highlight the importance of vibrational spectra acquisition from the region of interest (ROI) of dried sample droplet. This implies, for complete characterization of the sample, an equal number of vibrational spectra need to be collected from both the inner and outer-ring region of the dried sample droplet.

### 2.5. Influence of Biomolecule Differences in the Inner and Outer-Ring Regions of Dried Sample Drop for Patient Characterization

The analysis of plasma and serum samples from healthy donors indicated a difference in the distribution of the biomolecules (Figure 3 and Figure S6, ESI) in the inner and outer-ring regions of the dried sample droplet. To verify the influence of these biochemical differences present in the inner and outer-ring regions, and to further validate the applications of the proposed measurement schematic, two clinically relevant examples have been discussed in the following sections: Plasma and serum samples collected in a longitudinal study from (a) a heart failure (HF) patient (sample collected at three-time points) and (b) from ischemic cardiomyopathy (ICM) patient (sample collected at two-time points) in response to treatment. The details of the treatment and the patient outcome study are not the focus of the current study.

In the following sub-sections, it has been shown in the case of HF patient, the PCA analysis obtained for the Raman spectra collected from the inner and the outer-ring regions are similar, however for FT-IR inner region of the sample, the droplet is of importance. Whereas, in the case of the ICM patient, dissimilar results are obtained by applying a PCA model on the Raman and FT-IR spectra collected from the inner region and outer-ring region.

#### 2.5.1. A Heart Failure Patient

Heart failure is a complex syndrome in which the heart muscle cannot pump the blood efficiently to supply enough blood to the body. The HF patient undergoes regular clinic check-ups, where blood samples are routinely analyzed to monitor the patient’s condition. In the current study, plasma and serum samples were collected from one HF patient, at three different time points, recruited in an ongoing clinical study to monitor patients’ response to therapy.

**Figure 4:**
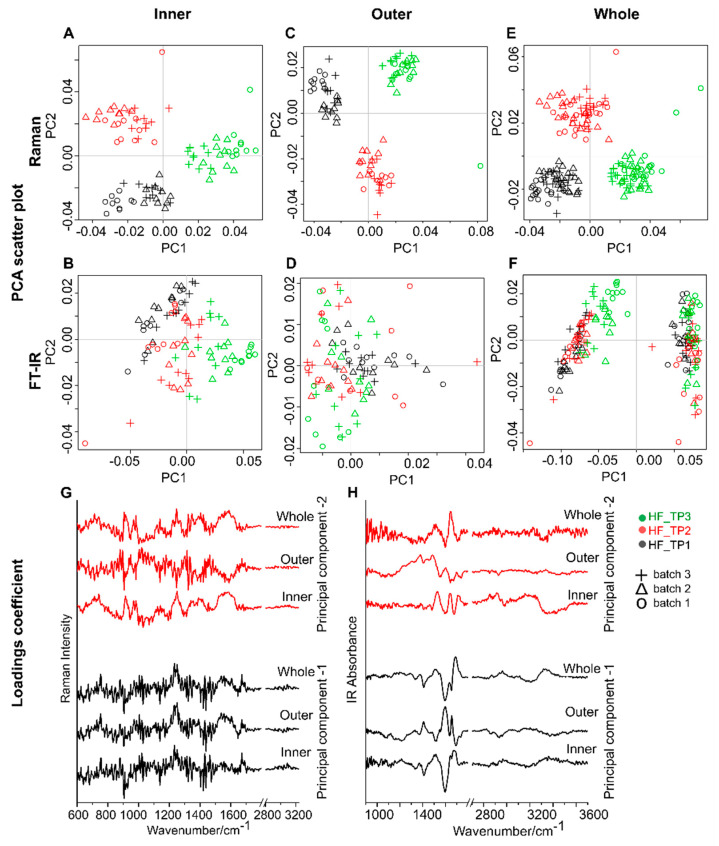
PCA analysis of Raman (first row) and FT-IR spectra (second row) collected from (**A**,**B**) inner region and (**C**,**D**) outer-ring regions from the plasma of the HF patient (*n* = 1). **(E**,**F**) combined analysis of the vibrational spectra collected from both inner and outer-ring regions. The principal components 1 and 2 of (**G**) Raman and (**H**) FT-IR data. The different time points (TP1 to TP3) of the patient sample collection are color coded, and the experimental replicates are presented as symbols (+, ∆ and o).

The current aim of the study was to follow biochemical changes occurring in the plasma and serum of the patient at the three different time points. Correlating patient outcome with the vibrational spectroscopy results is out of the scope of the current study. The main aim is to show difference in the results obtained while analyzing inner and outer-ring regions of the droplets. From the HF patient, plasma and serum samples collected at three different time points (Day 1, Day 5, and Day 60) were analyzed by applying the PCA method. The PCA was performed on the vibrational spectral (Raman and FT-IR) data collected from the inner and outer-ring regions of the dried plasma (Figure 4) and serum (Figure S7, ESI) samples.

As described in the Methods section, the analysis was done separately on the spectra collected from the inner region, outer-ring region, and combining the spectra from both inner and outer-ring regions. The PC score plot for the Raman data is shown in Figure 4A,C,E and the FT-IR data are shown in Figure 4B,D,F. The PCA loadings, PC-1 and PC-2, are displayed in Figure 4G (for the Raman data) and Figure 4H (for the FT-IR data). The ASCA model performed to analyze the design of our experiment and calculating the contribution of the experiment into overall variance is presented for the Raman and FT-IR spectral data set (Table 1 for plasma samples and Table S1, ESI for serum samples).

The PCA model fitted with a matrix estimated for each factor contribution has been displayed in Figure S8, ESI for the plasma samples and Figure S9, ESI for the serum samples. The obtained results showed that Raman spectra of plasma samples collected at three different time points indicate clear separation irrespective of the positions from where the spectra were collected (Figure 4A,C,E). The ASCA model confirms the Raman spectroscopy observation, where the factor “position” calculated for the Raman data has the lowest contribution, a variance of 4.22% (Table 1). Although, in the variance analysis, the “Batch” captures the highest variance indicating higher influence coming from the technical replication, the three sampling time points can, however, be separated (Figure S8A, ESI). These results indicate to monitor the chosen HF patient’s condition, and the Raman spectra of the plasma either from an inner region or from the outer-ring region of the dried sample droplet are enough. Similarly, for the serum sample time point, HF_TP3 can be well separated from the other two time points irrespective of the spectral acquisition position (Figure S7A,C,E, ESI). This is also confirmed from the ASCA-based analysis of variance (Table S1 (left), ESI). In the case of the FT-IR spectroscopy data, the PCA analysis for the FT-IR spectra of plasma from the HF patient, only the spectra from inner region show separation among the three different sampling time points (Figure 4B).

Neither the FT-IR spectra collected from outer-ring region nor the spectra collected from whole droplet (i.e., combined analysis of the spectra from the inner and outer-ring regions) can differentiate between. the three different time points (Figure 4D,F). This observation is further supported by the analysis of variance performed on the FT-IR spectra. As observed from Table 1, the factor “position” has the highest variance of 78.84%, indicating the position of the FT-IR spectra collection influences the characterization of the HF patient plasma collected at different time points. This is further supported by the factor “Time” in the ASCA model, that describes the experimental design, of the FT-IR data, which has the lowest contribution into the overall variance.

The analysis based on the PCA models and the ASCA of the vibrational spectra collected from the studied HF-patient can be summarized as mentioned below: The time points of blood sampling can be well differentiated using Raman spectral data, irrespective of the sample position from where the Raman spectra were acquired. However, while employing FT-IR spectroscopy for the investigation of plasma and serum sample from an HF patient, the sample position of FT-IR spectra features the highest contribution in the characterization of the sampling time points.

#### 2.5.2. An ischemic Cardiomyopathy Patient

**Figure 5:**
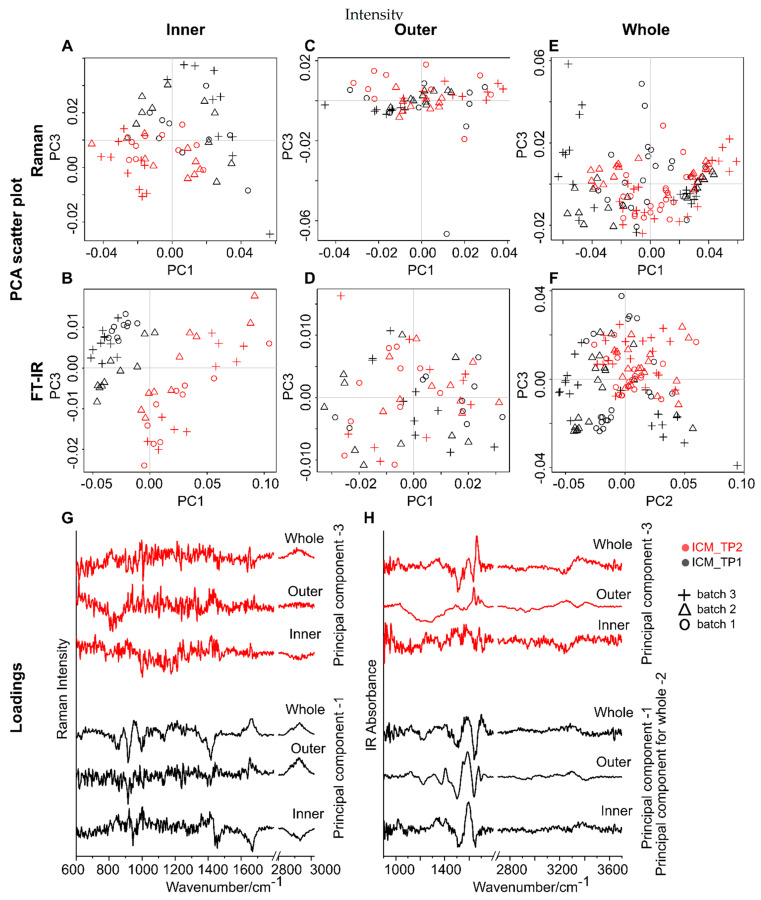
PCA analysis of Raman (first row) and FT-IR spectra (second row) collected from (**A**,**B**) inner region and (**C**,**D**) outer-ring regions from the plasma of the ICM patient (*n* = 1). (**E**,**F**) combined analysis of the vibrational spectra collected from both inner and outer-ring regions. The principal components 1 and 3 of (**G**) Raman and (**H**) FT-IR data. The different time points (TP1 and TP2) of the patient sample collection are color coded, and the experimental replicates are presented as symbols (+, ∆ and o).

The ischemic cardiomyopathy (ICM) condition is related to narrowing of the coronary arteries. As a second case study, an ICM patient recruited in the ongoing clinical trial was investigated. The aim was to explore the influence of sample position on the chemical information contained within the vibrational spectroscopy data recorded from the dried plasma and serum samples. The investigated plasma and serum samples were collected from the ICM patient at two different time points (before and one day after treatment). A similar analysis, as aforementioned for the HF patient, was performed to follow the plasma and serum composition changes in the ICM patient before and after treatment. The PCA score plots for the plasma samples and the serum samples are shown in Figure 5 and Figure S10, ESI, respectively.

Furthermore, the results of ASCA based on the considered experiment factors are shown in Table 2 for the plasma samples and in Table S2, ESI for the serum samples. The PCA analysis on the calculated factors is displayed in Figure S11, ESI for the plasma samples and in Figure S12, ESI for the serum samples.

Both Raman and FT-IR spectra of the plasma samples collected from the two time points show clear separation only when the spectra were collected from the inner region; see Figure 5A,B. The inner region of the dried droplet contributes to the treatment condition-related information (time-points) for this patient. The chemical information for the discrimination of the two-time points can be visualized in the PC loading plots (Figure 5G,H). Applying the analysis of variance confirms that the factor “Position” has a high contribution to the overall data variance for Raman spectra with a value of 16.06%. Likewise, for FT-IR spectra, the sample position contributed highly into the overall data variance with a value of 69.82% (Table 2). For serum samples, FT-IR spectra collected from either inner or outer-ring regions can differentiate the two-time points (Figure S10B,D, ESI). Whereas the Raman spectral data collected from serum samples do not distinguish the two-time points.

The FLIM images were acquired for plasma and serum samples of both the patients (Figure S13, ESI). Fluorescent lifetime analysis revealed differences in the lifetimes of the biomolecules from the inner and the outer-ring region. Fast FLIM images were depicted in pseudo-colored images, with blue representing the shorter lifetime, corresponding to the inner region for both serum and plasma samples, which was readily distinguishable from the longer lifetime observed in the periphery region (Figure S13, ESI). One can visualize the drying pattern and the difference in the biomolecular distribution, of the plasma and serum samples, between HF and the ICM patients. Although, at this point, it is hard to pinpoint the identity of the biomolecules contributing to the FLIM images. A detailed analysis of molecular composition of the plasma and serum samples of HF and ICM patients can shed more light on the observed differences in the FLIM images.

We further evaluated the grouping tendencies among the individuals with diverse health conditions: Healthy donors and cardiac patients, to visualize the influence of inner and outer-ring regions for respective group separation. In the supplementary Figures S13 and S14, PCA analysis for plasma and serum samples has been presented, respectively. For the Raman spectral data of plasma samples (Figure S14A,C,E, ESI) cardiac patients are closely clustered and can be distinguished from the healthy donors, especially, for the Raman spectral data acquired from the outer-ring region (Figure S14C, ESI) the separation is prominent. The healthy donors and patients’ group separation for the FT-IR data acquired from the plasma samples is not prominent (Figure S14B,D,F, ESI). The possible cause is high heterogeneity among healthy donors as seen in the PCA score plot (Figure S14, ESI). Similarly, the Raman spectral data collected from the inner and outer-ring regions of dried serum droplets show the good separation of healthy donors from the cardiac patients (Figure S15A,C, ESI). Whereas the combined Raman data from both inner and outer-ring regions (Whole) show considerable overlapping between both the groups (Figure S15E, ESI). In the case of the FT-IR serum data collected from the inner region of the dried droplet, two distinct clusters for the cardiac patients are seen, with PC2 separating patient CP01 from the patient CP02 (Figure S15B, ESI). However, there is considerable overlap with the healthy donors. Whereas for the outer-ring region, no separation is observed (Figure S15D, ESI). In contrast, from the combined FT-IR data from both inner and outer-regions of serum (Whole), a compact grouping of the cardiac patients is observed with PC1 and PC2, when considered together, separate the cardiac patients from the healthy donors (Figure S15F, ESI). Interestingly, the FT-IR serum spectra from the healthy donors’ cluster into two separate groups when the spectra collected from both inner and outer-ring regions are pooled together (Whole).

Although the healthy donors present large inter-group variations, the vibrational spectra collected from inner and outer-ring regions, when analyzed separately or pooled together, show distinct behavior during PCA analyses indicating a heterogeneous distribution of biochemical in the dried plasma/serum droplet.

Thus, the results presented for the two case examples support our hypothesis that when investigating dried droplet of plasma and serum samples via vibrational spectroscopy for clinical application, the sampling position is very important to deliver the clinical information. Hence, it cannot be taken for granted that the thick outer-ring formed due to the coffee-ring effect giving high-intensity spectra provides a complete characterization of the sample under investigation.

## 3. Experimental

### 3.1. Sample Collection and Preparation

Blood samples were collected from healthy donors and patients with compromised cardiac conditions (Figure S1. ESI). Informed consent as per the ethic committee (Ethic number 4736-04/16, 3558-08/12) from the Jena University Hospital was obtained before collecting the blood. Blood samples were collected using monovettes (SARSTEDT AG & Co. KG, Nimbrecht, Germany) with the anticoagulant ethylenediaminetetraacetic acid (EDTA) (for the study of plasma) and without anticoagulant (for the study of serum). Briefly, the blood samples were collected from 17 healthy donors, out of which 7 plasma samples and 10 serum samples were analyzed. Further, plasma and serum samples obtained from 2 patients were investigated. Both the patients were with compromised cardiac conditions, whereby one was diagnosed with heart failure (HF) and the second patient had ischemic cardiomyopathy (ICM). The blood samples from the HF patient were collected at three different time points, and the blood samples from the ICM patient were collected at two different time points. Blood samples were processed immediately after collection (~1 h) to obtain plasma and serum.

Plasma and serum were extracted by centrifugation method according to previously established [39] standard procedures routinely used in the clinic: Plasma samples were centrifuged for 10 min at 20 °C and 2000 relative centrifugal force (rcf), whereas, serum samples were centrifuged at 20 °C, 2500 rcf, and for 10 min. Aliquots of plasma and serum were stored at −80 °C until further use. The blood samples (plasma and serum) were prepared via drop coating deposition method, where 1μL of the sample was placed on a CaF_2_ slide (Crystal, Germany) and dried under the sterile bench for ~30 min. The samples were immediately investigated upon drying by Raman and FT-IR spectroscopy. For FT-IR spectroscopic investigation, the samples were diluted 1:10 with ultra-pure water (Ampuwa, KabiPac) to avoid the saturation of the FT-IR signal. As a reference substance, the most abundant blood protein albumin was used. Commercial albumin powder (from bovine serum, Merck) was dissolved in ultra-pure water (Ampuwa, KabiPac) and D_2_O (Sigma Aldrich, St. Louis, MO, USA) with a final concentration of 40mg/mL. The albumin dissolved in D_2_O was kept at 4 °C for one week for exchange of albumin C-H to C-D. Reference samples were prepared in the same way as the blood samples by drop coating deposition. To check the location of albumin in the dried plasma and serum droplets, albumin dissolved in D_2_O was mixed with plasma and serum in a ratio of 2:1 for Raman spectroscopy and mixed with pre-diluted (using ultra-pure water) plasma and serum in the same ratio for FT-IR spectroscopy. The mixture of deuterated albumin and plasma/serum was investigated immediately to avoid deuteration of plasma/serum components. The 3D-image of the dried plasma droplet under white light was captured using 405nm diode laser-guided via 60× objective with a numerical aperture (NA) of 0.75 (Zeiss, Germany) on the sample and the transmitted light detected with a photomultiplier tube (T-PMT) mounted on a confocal laser scanning microscope (CLSM) (LSM 780, Zeiss, Germany). The FIJI ImageJ platform was used to read the CZI data generated by the CLSM and to display the images.

### 3.2. Vibrational Spectroscopy Measurements

For Raman measurement, 785 nm laser with power of 175 mW in the sample plane was used. Raman spectra were collected in a back-scattered geometry with 2s exposure time via a 20×/0.8 NA objective (420650-9901, Plan-Apochromat, Zeiss, Germany). The collected spectra were guided via 100µm optical fiber to the grating-based (300 g/mm) spectrometer (UHTS 300, WITec, Germany) equipped with a CCD camera. FT-IR spectra were recorded by Mercury-Cadmium-Telluride (MCT) detector in transmission mode covering the spectral region of 900–3900 cm^−1^. The samples coated on a CaF_2_ slide were placed on the scanning stage of the imaging microscope (Cary 620 FT-IR, Agilent, Santa Clara, CA, USA), and an imaging aperture size of 50 µm × 50 µm was focused through a 25× objective with NA of 0.81. The FT-IR spectra of the samples were recorded with the spectral resolution of 4 cm^−1^ and each spectrum was an average of 16 scans (Cary 670FT-IR, Agilent, Santa Clara, CA, USA). To compensate for the contribution coming from atmospheric air, background scans were recorded on the sample-free substrate for each sample before the collection of FT-IR spectra.

To consider the heterogeneities of biomolecules’ distribution in the sample due to coffee-ring effect, Raman spectra were collected from 20 positions as shown in Figure S2A,B, ESI: 10 from the central zone (inner) and 10 from the droplet edge (outer-ring) for the samples from both healthy donors and the patients. For each sample, 3 spectra from each position were acquired. The FT-IR data from the patients’ serum and plasma samples were collected using the same measurement schematic as for the Raman data. Whereas, from the healthy donors’ plasma and serum samples, 10 random positions, 5 from the inner region, and 5 from the outer ring were recorded. For albumin studies, Raman and FT-IR spectra from 20 random positions on the dried droplet were collected.

For Raman spectroscopy imaging, Raman spectra were collected with a step size of 3 µm and integration time of 2 s. The FT-IR imaging of the samples was done using the same FT-IR spectrometer, however with a 64x64 focal-plane array (FPA) detector with an aperture size of 211 µm × 211 µm.

### 3.3. Fluorescence Lifetime Measurements

The blood samples (plasma and serum) were prepared in a similar way as described above. The dried sample on a CaF_2_ slide was analyzed using Leica TCS SP8 X Laser-scanning microscope (Leica Microsystems, Germany) using a HC PL APO CS2 20×/0.75 DRY objective. FLIM measurements were performed with a pulsed white light laser at 80 MHz pulse repetition rate with an excitation wavelength of 540 nm and detection of emission photons for fluorescent signals by the internal hybrid photon counting detector (HyD SMD 2) in the wavelength range of 560–800 nm. A PMT detector was also used simultaneously at a detection range of 380 nm–560 nm. Tile scans of the samples were recorded by using the following data acquisition parameters: Pixel dwell time of 1.58 μs, resolution of 1024 × 1024 pixels, scan speed of 400 Hz, accumulation of 10 frames, and an average of 2 scans per line.

The acquired FLIM images were analyzed with the LAS X FLIM/FCS software (Leica Microsystems, Germany). The lifetime analysis was carried out by selecting n-Exponential Reconvolution as fit model and fitting two exponential components with a fitting range of 0.048–12.461 ns. This fits pixel-by-pixel the fluorescence decay from all pixels in the image. The FLIM image was fitted using a threshold of 50 and a binning factor of 1, and two images were obtained corresponding to the different lifetime components.

### 3.4. Data Analysis

The raw spectral data were analyzed with a home-built algorithm using GUN R environment [44]. This included Raman and FT-IR spectra pre-processing, principal component analysis (PCA) [45], and the analysis of variance (ANOVA) based on ANOVA-simultaneous component analysis (ASCA) [46,47]. The PCA is a dimension reduction technique that aims to increase data interpretability by projecting a considered data set on a new uncorrelated variable space. For the PCA analysis, the data were mean-centered and the R function “prcomp(data)” was utilized, where “data” is the spectra matrix. The ASCA is an explorative tool that can be utilized to analyze the variance in multifactorial experimental designs when the measurements are described by multiple features i.e., multivariate measurements. Based on ASCA, a data matrix is decomposed into different effects refer to experiment factors and the interactions between these factors e.g., experiment methodology, biomolecule concentration, methods to collect the sample, or patient heterogeneity.

For Raman spectra, the pre-processing workflow involves removal of cosmic spikes, wavenumber calibration [48], baseline correction via asymmetric least squares [49], model transfer via extended multiplicative signal correction (EMSC) [50], spectral region selection (600–1800 cm^−1^ and 2800–3020 cm^−1^), and vector normalization. For FT-IR data, pre-processing steps are baseline correction using sensitive nonlinear iterative peak (SNIP) algorithm [48] followed by spectral region selection (900–1800 cm^−1^ and 2700–3700 cm^−1^) and vector normalization. After spectral pre-processing, three different PCA models were performed on spectra of the dried droplet: First collected from the inner region, the second model used the collected spectra from ring region, and the third model was based on Raman or FT-IR spectra acquired from both inner and outer-ring regions of the dried droplet. Furthermore, ASCA models were applied to the Raman spectroscopy data and to the FT-IR spectroscopy data to dig out the influence of different experiment factors on spectral datasets concerning the two patients. The aforementioned ASCA model was accomplished by estimating each spectral matrix by different terms representing the experiment factors. Then, a factor contribution is calculated based on the percentage of variance explained by this factor compared to the overall dataset variance. Lastly, a PCA model was utilized to interpret and visualize the analysis of variance results concerning each factor. In our study, the technical replicates (batches) with three experimental repetitions, the time for sample collection (Time) with two-time points, and the position of collected spectra (inner and outer-ring regions) formed the experiment factors. If **X** denotes a Raman or FT-IR spectral matrix of a specific patient, the ASCA model that describes our study can be formulated matrix for considering the experiment factors as following Equation (1):

(1) $$X=M_{0}+Con_{batch}+Con_{Time}+Con_{Position}+Ȇ$$

where M_0_ denotes a matrix in which mean Raman or FT-IR spectra with respect to the wavenumbers are oriented in rows, Con*_f_* represents a matrix of factor *f* Є {Batch, Time, Position}, and Ȇ is the residual matrix. This residual matrix indicates the estimation error of X and it usually describes the uncontrolled or unconsidered experiment factors in an explicitly designed study. The estimation of each factor matrix is uniquely achieved as described in [46,47]. Briefly, a matrix of factor ***f*** is approximated by the mean levels of this factor with respect to the wavenumbers.

## 4. Conclusions

A dried plasma or serum sample on a solid substrate has heterogenous distribution of biomolecules at the inner and the outer-ring regions. For the real-world biomedical applications, the disease specific biomarkers’ distribution patterns and the location in a dried sample varies for patients with different disease etiology. In this study, vibrational spectroscopy and fluorescence lifetime imaging has been applied to illustrate importance of the sample region at which the dried plasma and serum droplet is investigated to extract the clinical information from two patients with different cardiological conditions. Firstly, using deuterated albumin and plasma from healthy donors, we show that during sample preparation via drop coating method the outer-ring region of the dried droplet has high abundance of biomolecules and is rich in protein biomolecules. Secondly, Raman and FT-IR spectroscopy data combined with principal component and ANOVA-simultaneous component analyses show biochemical difference between the inner region and the outer-ring region of the dried sample droplet. These biochemical differences have been further supported by FLIM analysis where different lifetimes were observed for the molecules located in the inner and the outer-ring regions of the dried sample droplet. Hence, the measurement schematic of equal sampling positions from inner and outer-ring regions allows capturing the information on different biomolecules in the whole sample droplet. Further, the segregated analysis of the inner and outer-ring region of the dried sample droplet provides a better overview of the contribution of the biomolecules for patient stratification, and eventually the efficacy of the treatment can be studied.

## Figures and Tables

**Table 1:** Variance analysis of different factors from the plasma of the HF patient (*n* = 1).

	Factors	Batches	Time	Position	Residuals
Variance					
Raman (in %)		23.74	23.69	4.22	48.36
FT-IR (in %)		0.77	3.53	78.84	16.86

**Table 2:** Variance analysis of different factors from the plasma of the ischemic cardiomyopathy (ICM) patient (*n* = 1).

	Factors	Batches	Time	Position	Residuals
Variance					
Raman (in %)		25.80	2.91	16.06	55.23
FT-IR (in %)		1.42	3.21	69.82	25.55

## Data Availability

Not applicable.

## Supplementary Materials

The following are available online at https://www.mdpi.com/1422-0067/22/4/2191/s1.
