# Supplementary material for: Vibrational Spectroscopic Investigation of Blood Plasma and Serum by Drop Coating Deposition for Clinical Application
Source: Int J Mol Sci. 2021 Feb 22;22(4):2191. doi: 10.3390/ijms22042191 (PMC7926873; doi:10.3390/ijms22042191)
Supplement: Supplementary file 1 [file ijms-22-02191-s001.pdf]

Supplementary information

Article

Vibrational spectroscopic investigation of blood plasma and serum by drop coating deposition for clinical application

Jing Huang [1,2], Nairveen Ali [1,2], Elsie Quansah [1,2], Shuxia Guo [1,2], Michel Noutsias [3,4], Tobias Meyer-Zedler [1,2], Thomas Bocklitz [1,2], Jürgen Popp [1,2,5,6], Ute Neugebauer [1,2,5,6] and Anuradha Ramoji [1,2,5,*]

1. Institute of Physical Chemistry and Abbe Center of Photonics, Friedrich-Schiller-University, Helmholtzweg 4, D-07743 Jena, Germany; jing_huang@fudan.edu.cn (J.H.); nairveen.ali@uni-jena.de (N.A.); elsie.quansah@uni-jena.de (E.Q.); shuxia_guo@seu.edu.cn (S.G.); tobias.meyer@leibniz-ipht.de (T.M.-Z.); thomas.bocklitz@uni-jena.de (T.B.); juergen.popp@uni-jena.de (J.P.); ute.neugebauer@med.uni-jena.de (U.N.)
2. Leibniz Institute of Photonic Technology, Member of Leibniz Health Technologies, Albert-Einstein-Straße 9, D-07745 Jena, Germany.
3. Department of Cardiology Internal Medicine, Jena University Hospital, Am Klinikum 1, D-07747 Jena, Germany; michel.noutsias@uk-halle.de
4. Mid-German Heart Center, Department of Internal Medicine III (KIM-III), Division of Cardiology, Angiology and Intensive Medical Care, University Hospital Halle, Martin-Luther-University Halle-Wittenberg, Ernst-Grube-Strasse 40, D-06120 Halle (Saale), Germany.
5. Center for Sepsis Control and Care (CSCC); Jena University Hospital, Am Klinikum 1, D-07747 Jena, Germany.
6. InfectoGnostics Research Campus Jena, Centre of Applied Research, Philosophenweg 7, D-07743 Jena, Germany.

* Correspondence: anuradha.ramoji@uni-jena.de; Tel.: +49(0)3641-9390926

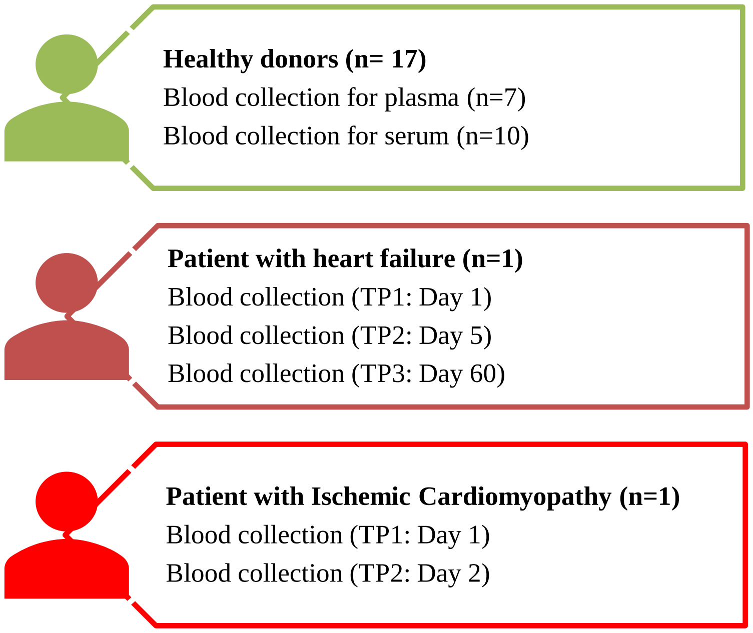

Figure S1. Details of the samples from the healthy donors and the patients investigated using vibrational spectroscopy.

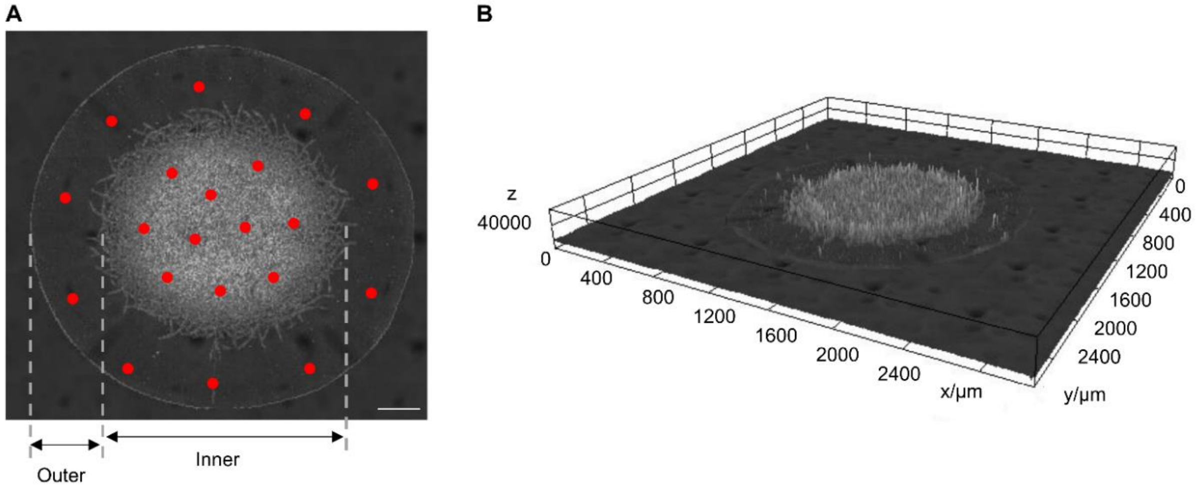
A
B
Outer
Inner
z
40000
0
400
800
1200
1600
2000
2400
x/μm
y/μm

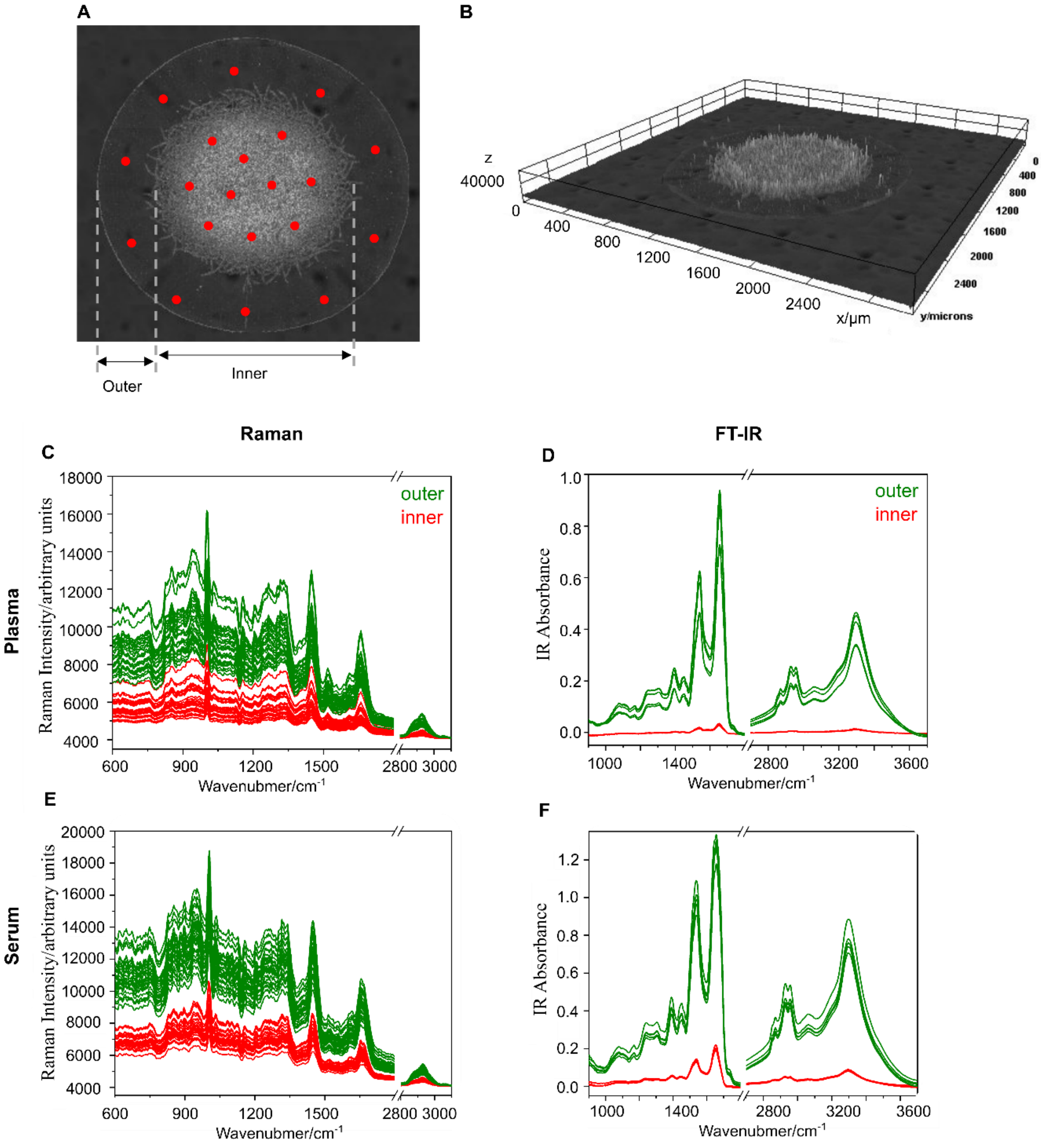
A
B
Outer
Inner
z
40000
0
x/μm
y/microns
Raman
FT-IR
C
D
E
F
Plasma
Serum
outer
inner
Raman Intensity/arbitrary units
IR Absorbance
Wavenubmer/cm-1

Figure S2. Representative image of pattern of a dried sample along and the vibrational spectra collected from different region of the dried droplet. (**A**) 2D-image showing data collection position from inner and outer region of the dried plasma sample spot of a healthy donor, (**B**) 3D-image visualizing the crystal pattern formed in the inner region of the dried sample. Scale bar is 200μm. Unprocessed Raman spectra collected from different regions of the dried (**C**) plasma and (**E**) serum droplet. (Healthy donor, *n* = 1). Unprocessed FT-IR spectra collected from different region of the dried (**D**) plasma and (**F**) serum droplet. The vibrational spectra collected from the inner region are depicted in red and spectra from the outer-ring region in green.

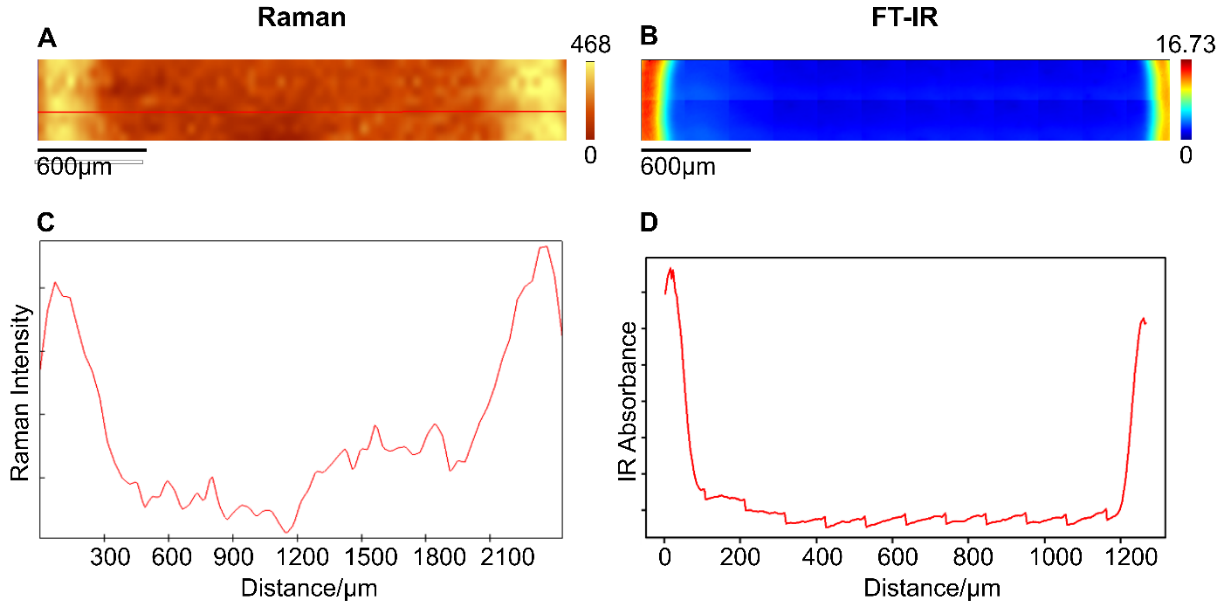

Figure S3. False colour (**A**) Raman image and (**B**) FT-IR image of the dried serum droplet showing intensity distribution of amide I vibrations around 1660cm^{-1} along with (**C**) Raman and (**D**) FT-IR intensity profile from the transverse axis.

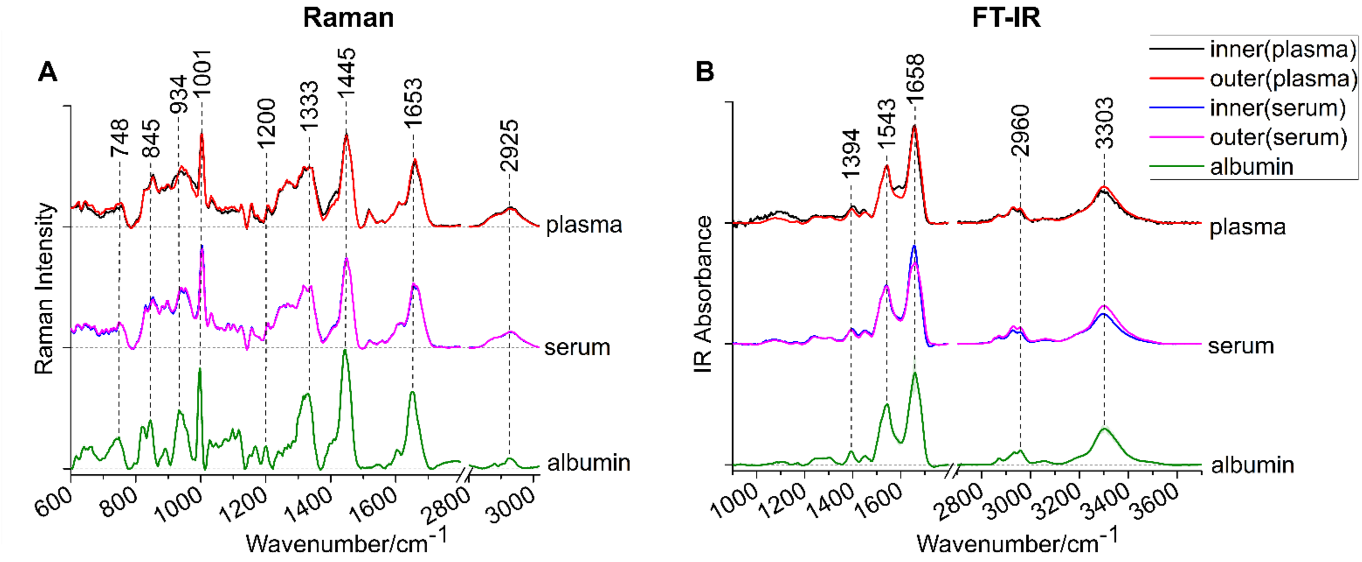

Figure S4. Pre-processed average (**A**) Raman and (**B**) FT-IR spectra of plasma, serum and albumin from inner and outer-ring regions. The spectra have been shifted along y-axis for clarity.

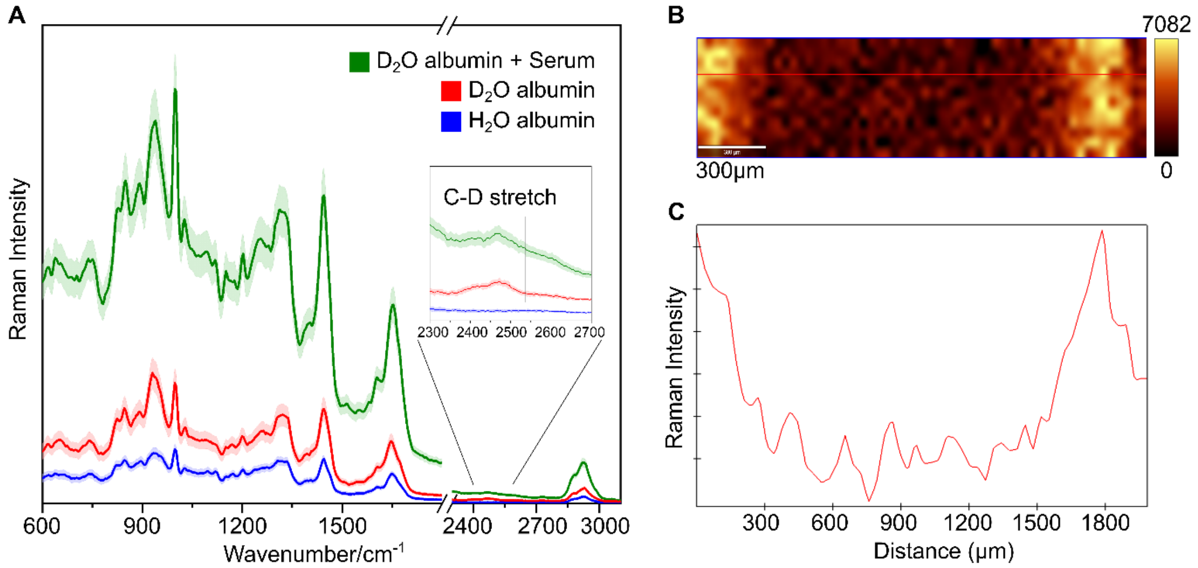

Figure S5. (**A**) Raman spectra comparison of deuterated (D_2O albumin), non-deuterated albumin (H_2O albumin) and deuterated albumin mixed with serum of a healthy donor in the ratio 1:2 (D_2O albumin + Serum). Inset shows enlarged Raman spectral region around 2300 cm^{-1} to 2700cm^{-1}. (**B**) False colour Raman images of the dried D_2O-albumin + Serum showing intensity distribution of C-D stretching vibration around 2470 cm^{-1} along with (**C**) the intensity profile from the transverse axis.

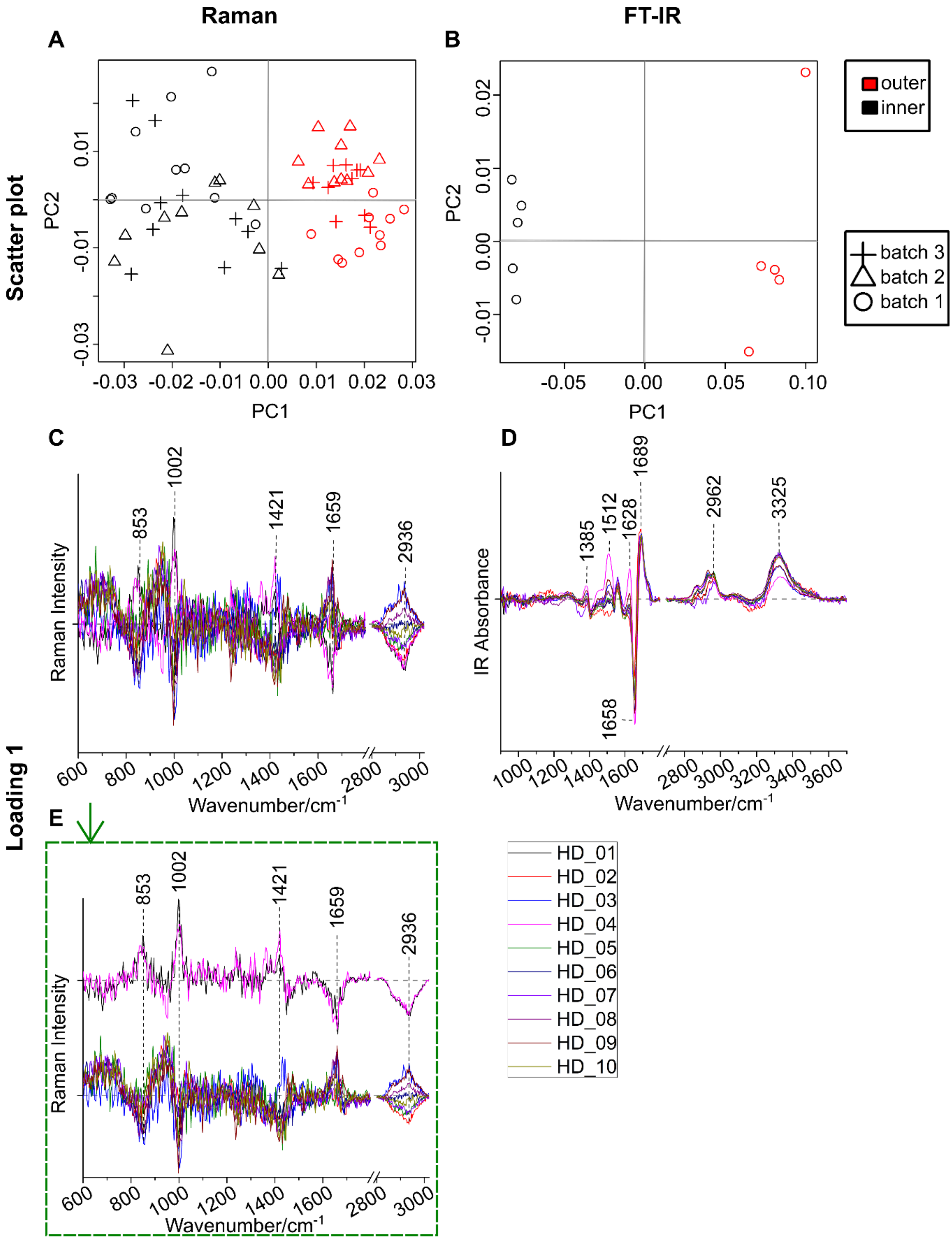

Figure S6. Principal component analysis (PCA) analysis (A) Raman spectra and (B) FT-IR spectra collected from inner and outer regions of the dried serum droplet from a healthy donor (n=1). PCA analysis was performed separately for n=10 healthy donors for the sake of space only the loadings coefficient PC1 for (C) Raman and (D) FT-IR spectral data has been shown for different healthy donors obtained. The contributing Raman bands captured by the PC1 is not similar for all the donors. For sake of clarity in Figure S5E, PC1 has been re-plotted in two groups according to their spectral features. These differences in PC1 obtained for serum samples correlates to the PCA scores obtained for the two donors: HD_01 and HD_04. For these two donors the composition of biomolecules present in the inner and outer regions of the serum droplet shows opposite trends compared to other donors.

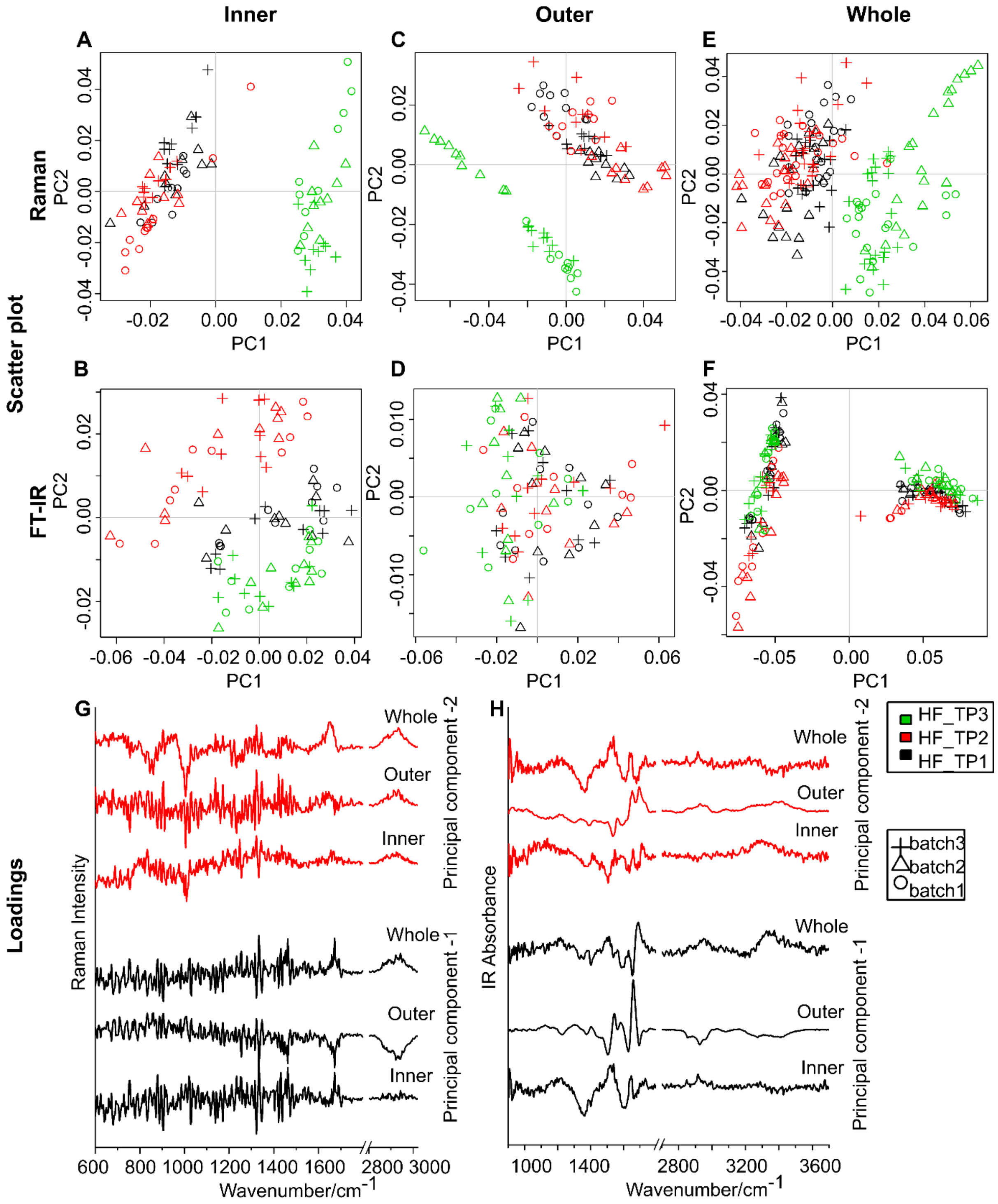

Figure S7. PCA analysis of Raman (first row) and FT-IR spectra (second row) collected from (**A** and **B**) inner region and (**C** and **D**) outer regions from the serum of the HF patient (n = 1). (**E** and **F**) combined analysis of the vibrational spectra collected from both inner and outer region. The principal components 1 and 2 of **G**) Raman and (**H**) FT-IR data. The different time points (TP1 to TP3) of the patient sample collection is colour coded and the experimental replicates are presented by the symbols (+, Δ and o).

Table S1. Variance analysis of different factors from the serum of the HF patient (n = 1).

	**Raman**				**FT-IR**			
**Factors**	**Batches**	**Time**	**Position**	**Residuals**	Batches	Time	Position	Residuals
**Variance (in %)**	27.96%	14.23%	6.79%	51.02%	0.72%	3.18%	75.44%	20.67%

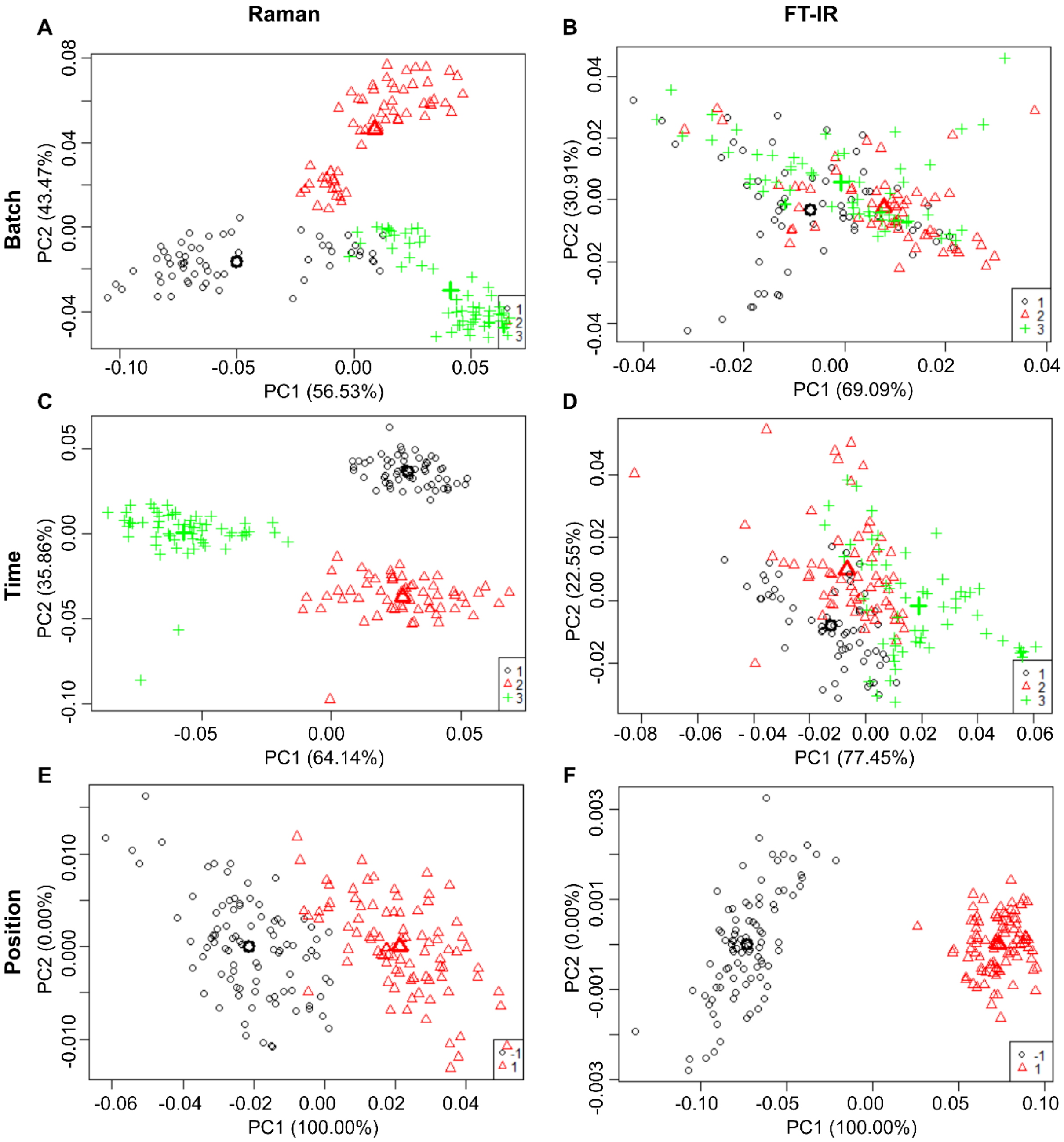

Figure S8. PCA scatter plot of different experimental factors influencing the spectral dataset, calculated using ASCA models applied to the vibrational spectra collected from the plasma sample of HF patient. PCA scatter plot of factor "Batch" calculated for (**A**) Raman spectra and (**B**) FT-IR spectra. PCA scatter plot of factor "Time" calculated for (**C**) Raman spectra and (**D**) FT-IR spectra . PCA scatter plot for factor "Position" calculated for (**E**) Raman spectra and (**F**) FT-IR spectra. The bold legend shows the mean of the group in the respective PCA scatterplot. In panels (**A** and **B**), the experimental replicates are presented by the symbols (o, Δ and +) and colour coded (black-batch1, red-batch2 and green-batch3). In the panel (**C** and **D**), the sampling time points are presented by the symbols (o, Δ and +) and the colour coding (black-TP1, red-TP2 and green-TP3). In the panels (**E** and **F**) the position of spectra acquired from the sample are presented by the symbols (o and Δ) and colour coding (black-inner region and red-outer region).

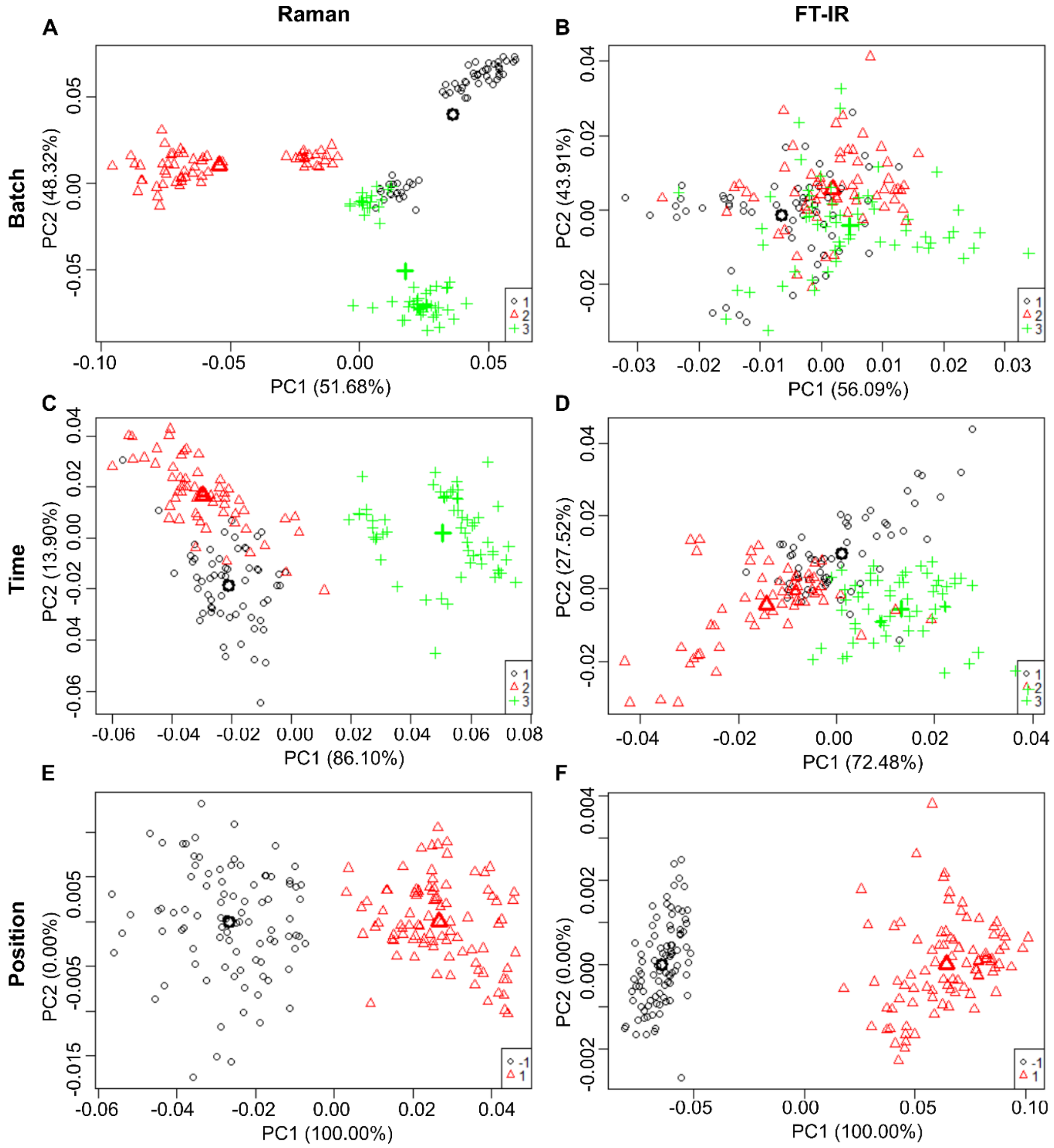

Figure S9. PCA scatter plot of different experimental factors influencing the spectral dataset, calculated using ASCA models applied to the vibrational spectra collected from the serum sample of HF patient. PCA scatter plot of factor "Batch" calculated for (**A**) Raman spectra and (**B**) FT-IR spectra. PCA scatter plot of factor "Time" calculated for (**C**) Raman spectra and (**D**) FT-IR spectra . PCA scatter plot for factor "Position" calculated for (**E**) Raman spectra and (**F**) FT-IR spectra. The bold legend shows the mean of the group in the respective PCA scatterplot. In panels (**A** and **B**), the experimental replicates are presented by the symbols (o, Δ and +) and colour coded (black-batch1, red-batch2 and green-batch3). In the panel (**C** and **D**), the sampling time points are presented by the symbols (o, Δ and +) and the colour coding (black-TP1, red-TP2 and green-TP3). In the panels (**E** and **F**) the position of spectra acquired from the sample are presented by the symbols (o and Δ) and colour coding (black-inner region and red-outer region).

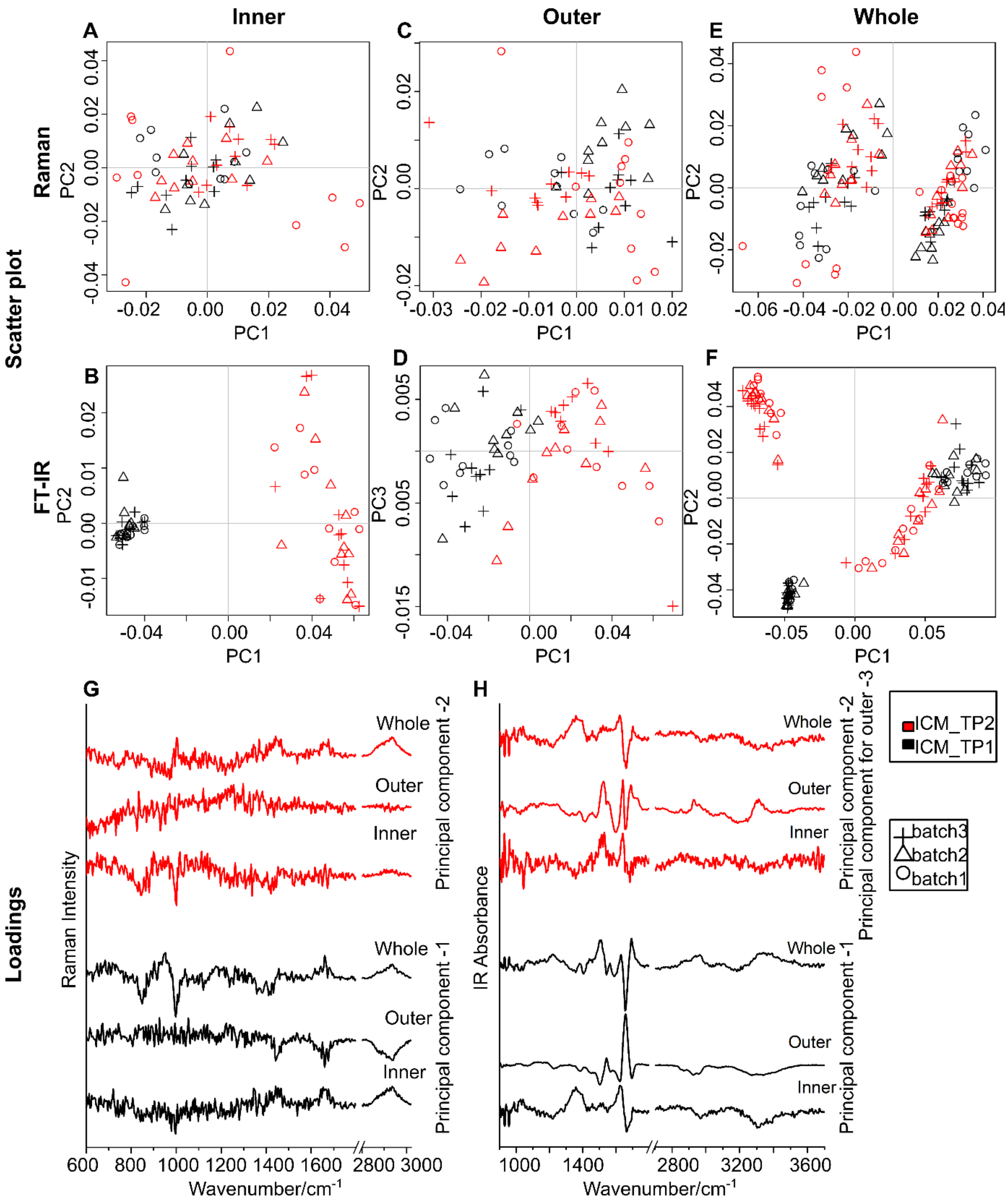

Figure 10. PCA analysis of Raman (first row) and FT-IR spectra (second row) collected from (**A** and **B**) inner region and (**C** and **D**) outer regions from the plasma of the ICM patient ($n = 1$). (**E** and **F**) combined analysis of the vibrational spectra collected from both inner and outer region. The principal components (PC) (**G**) for Raman data PC-1 and PC-2 and (**H**) for FT-IR data, PC-1 and PC-2 (inner and whole) PC-3 (outer region) . The different time points (TP1 and TP2) of the patient sample collection is colour coded and the experimental replicates are presented by the symbols (+, Δ and o).

Table S2. Variance analysis of different factors from the serum of the ICM patient ($n = 1$).

	**Raman**				**FT-IR**			
**Factors**	**Batches**	**Time**	**Position**	**Residuals**	Batches	Time	Position	Residuals
**Variance (in %)**	30.54%	1.36%	20.50%	47.61%	1.07%	10.59%	57.01%	31.34%

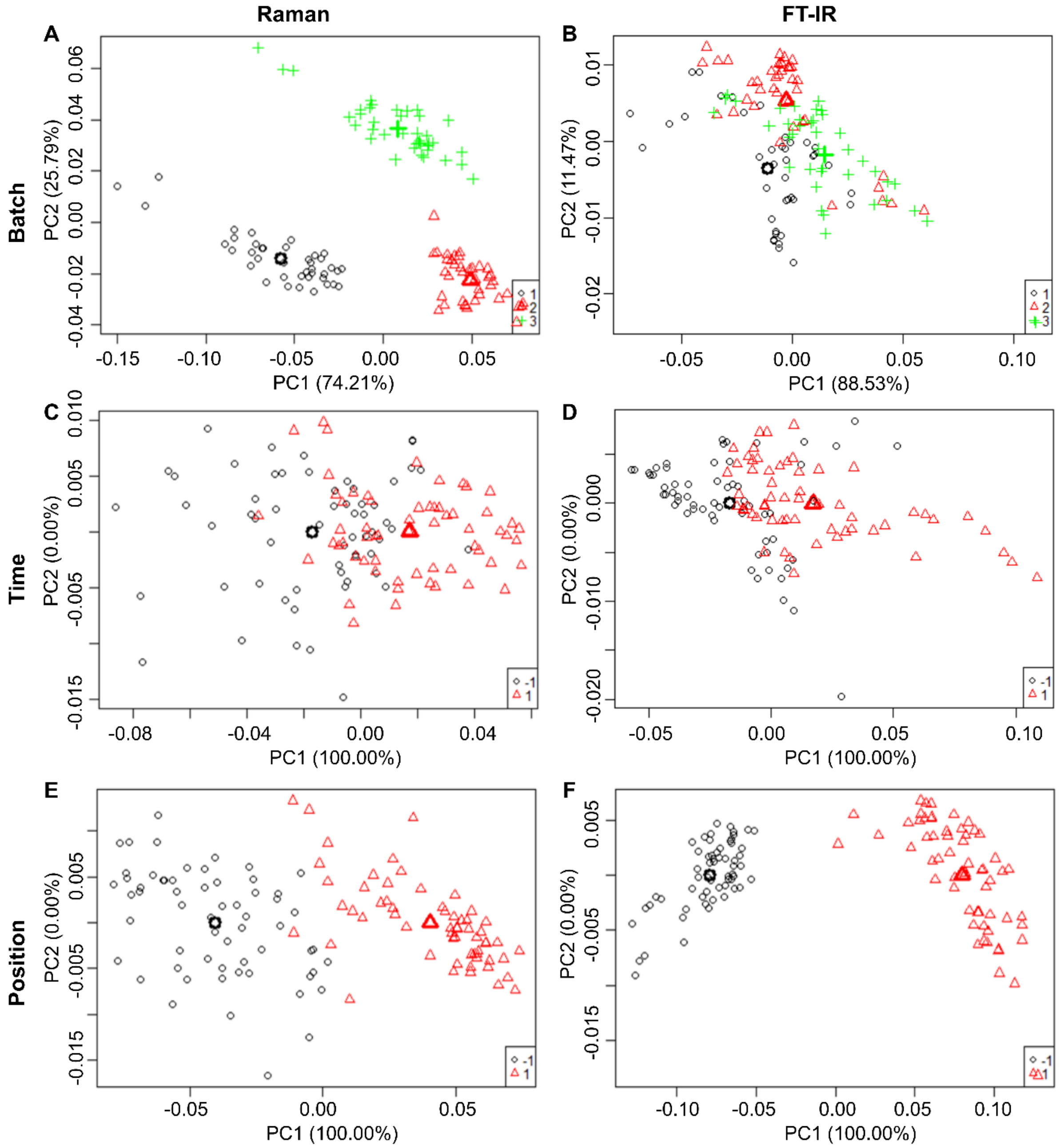

Figure S11. PCA scatter plot of different experimental factors influencing the spectral dataset, calculated using ASCA models applied to the vibrational spectra collected from the plasma sample of ICM patient. PCA scatter plot of factor "Batch" calculated for (**A**) Raman spectra and (**B**) FT-IR spectra. PCA scatter plot of factor "Time" calculated for (**C**) Raman spectra and (**D**) FT-IR spectra . PCA scatter plot for factor "Position" calculated for (**E**) Raman spectra and (**F**) FT-IR spectra. The bold legend shows the mean of the group in the respective PCA scatterplot. In panels (**A** and **B**), the experimental replicates are presented by the symbols (o, Δ and +) and colour coded (black-batch1, red-batch2 and green-batch3). In the panel (**C** and **D**), the sampling time points are presented by the symbols (o and Δ) and the colour coding (black-TP1 and red-TP2). In the panels (**E** and **F**) the position of spectra acquired from the sample are presented by the symbols (o and Δ) and colour coding (black-inner region and red-outer region).

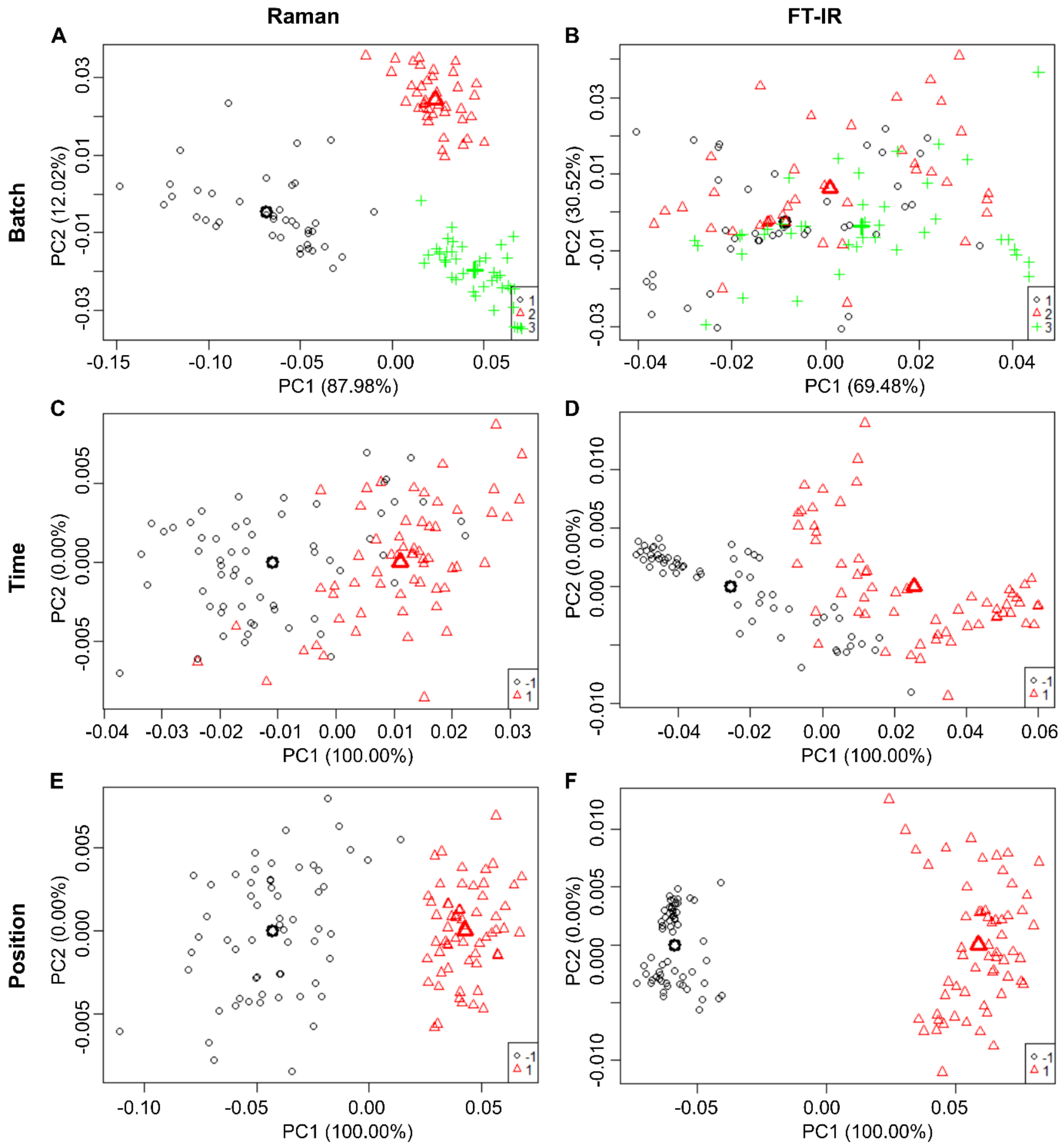

Figure S12. PCA scatter plot of different experimental factors influencing the spectral dataset, calculated using ASCA models applied to the vibrational spectra collected from the serum sample of ICM patient. PCA scatter plot of factor “Batch” calculated for (**A**) Raman spectra and (**B**) FT-IR spectra. PCA scatter plot of factor “Time” calculated for (**C**) Raman spectra and (**D**) FT-IR spectra . PCA scatter plot for factor “Position” calculated for (**E**) Raman spectra and (**F**) FT-IR spectra. The bold legend shows the mean of the group in the respective PCA scatterplot. In panels (**A** and **B**), the experimental replicates are presented by the symbols (o, Δ and +) and colour coded (black-batch1, red-batch2 and green-batch3). In the panel (**C** and **D**), the sampling time points are presented by the symbols (o and Δ) and the colour coding (black-TP1 and red-TP2). In the panels (**E** and **F**) the position of spectra acquired from the sample are presented by the symbols (o and Δ) and colour coding (black-inner region and red-outer region).

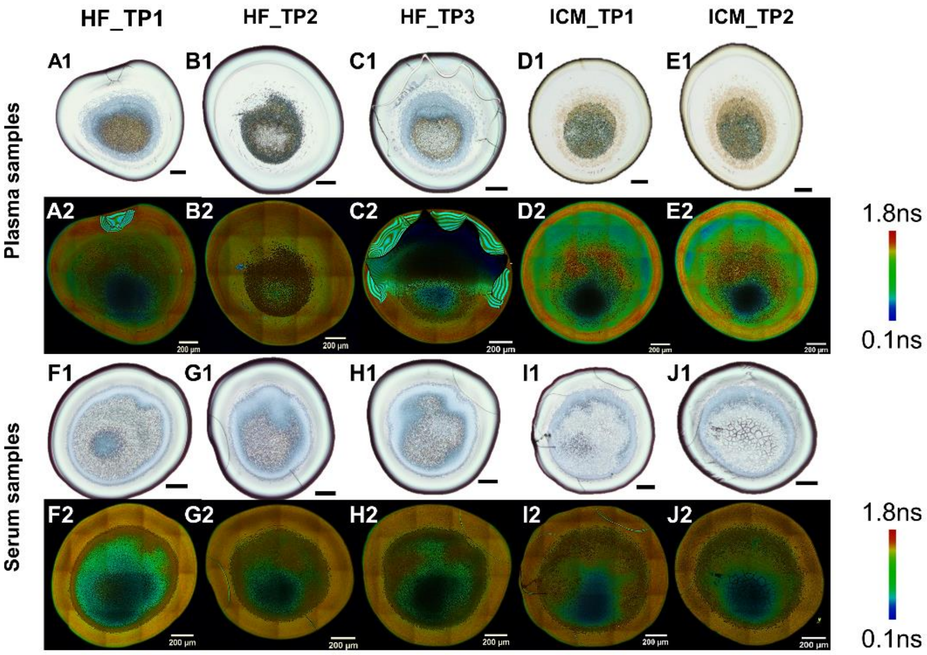

Figure S13. Fluorescence lifetime images of dried plasma and serum droplet from HF and ICM patients. (**A1, B1, C1**,) white light images and (**A2, B2, C2**) FLIM image of HF patient's plasma sample and (**F1, G1, H1**) white light images and (**F2, G2, H2**) FLIM images of HF patient's serum sample. (**D1, E1**) white light images and (**D2, E2**) FLIM image of ICM patient's plasma sample and (I1, J1) white light images and (**I2, J2**) FLIM images of ICM patient's serum sample. The color-coded lifetime values are from 0.1–1.8 ns. Scale bar is 200 μm.

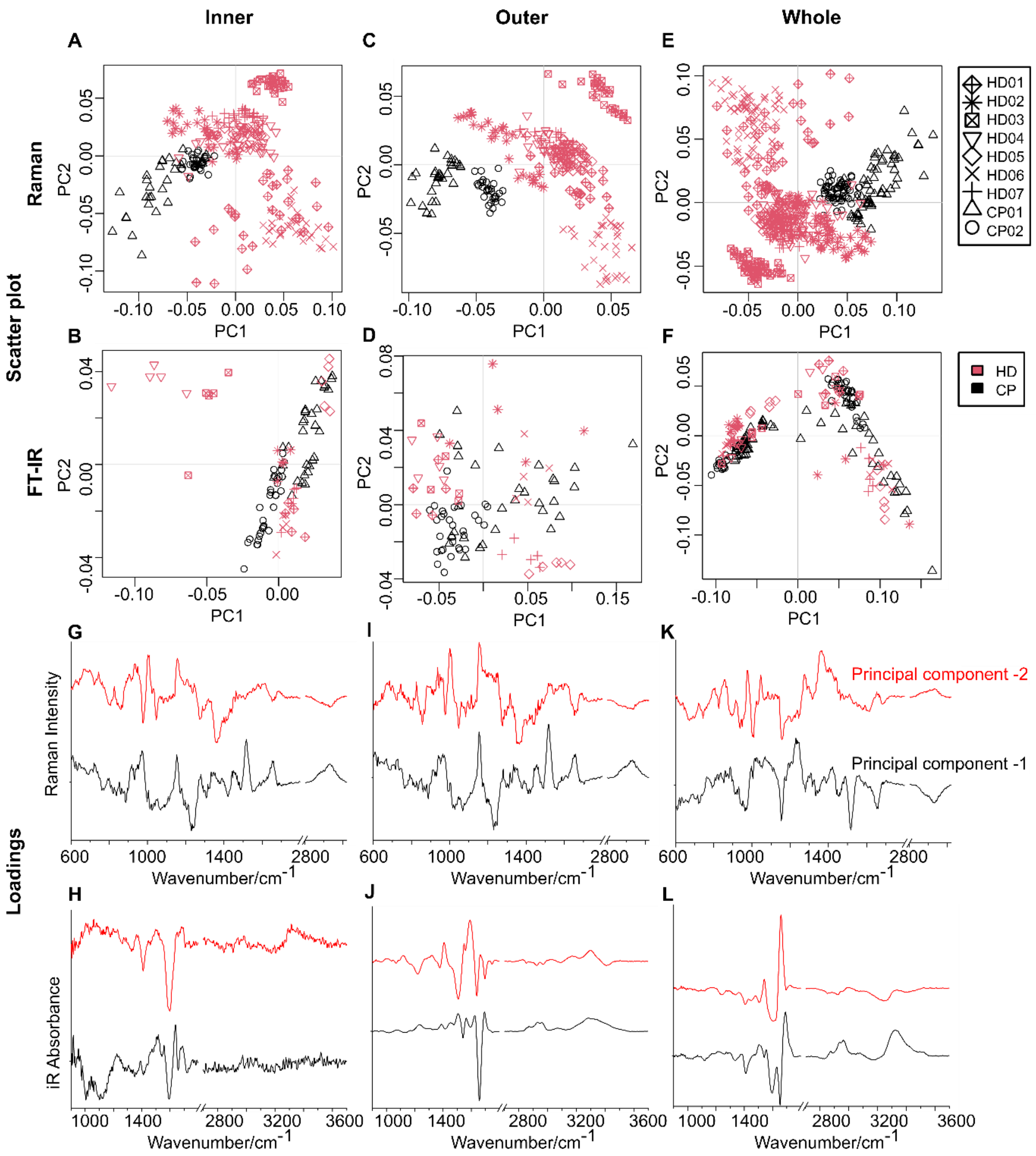

Figure S14. PCA grouping tendencies between healthy donors and cardiac patients calculated using vibrational spectroscopy data from dried plasma sample droplet. PCA analysis of Raman (first row) and FT-IR spectra (second row) collected from (**A** and **B**) inner region and (**C** and **D**) outer regions from the dried plasma sample droplet for nine different healthy donors (red color and individual donors are denoted by symbols) and two cardiac patients: CP01 is ICM patient and CP02 is HF patient (black color and individual cardiac patients are denoted by symbols). (**E** and **F**) combined analysis of the vibrational spectra collected from both inner and outer region. The principal components 1 (black) and 2 (red) for Raman (**G, I** and **K**) and FT-IR data (**H, J** and **L**).

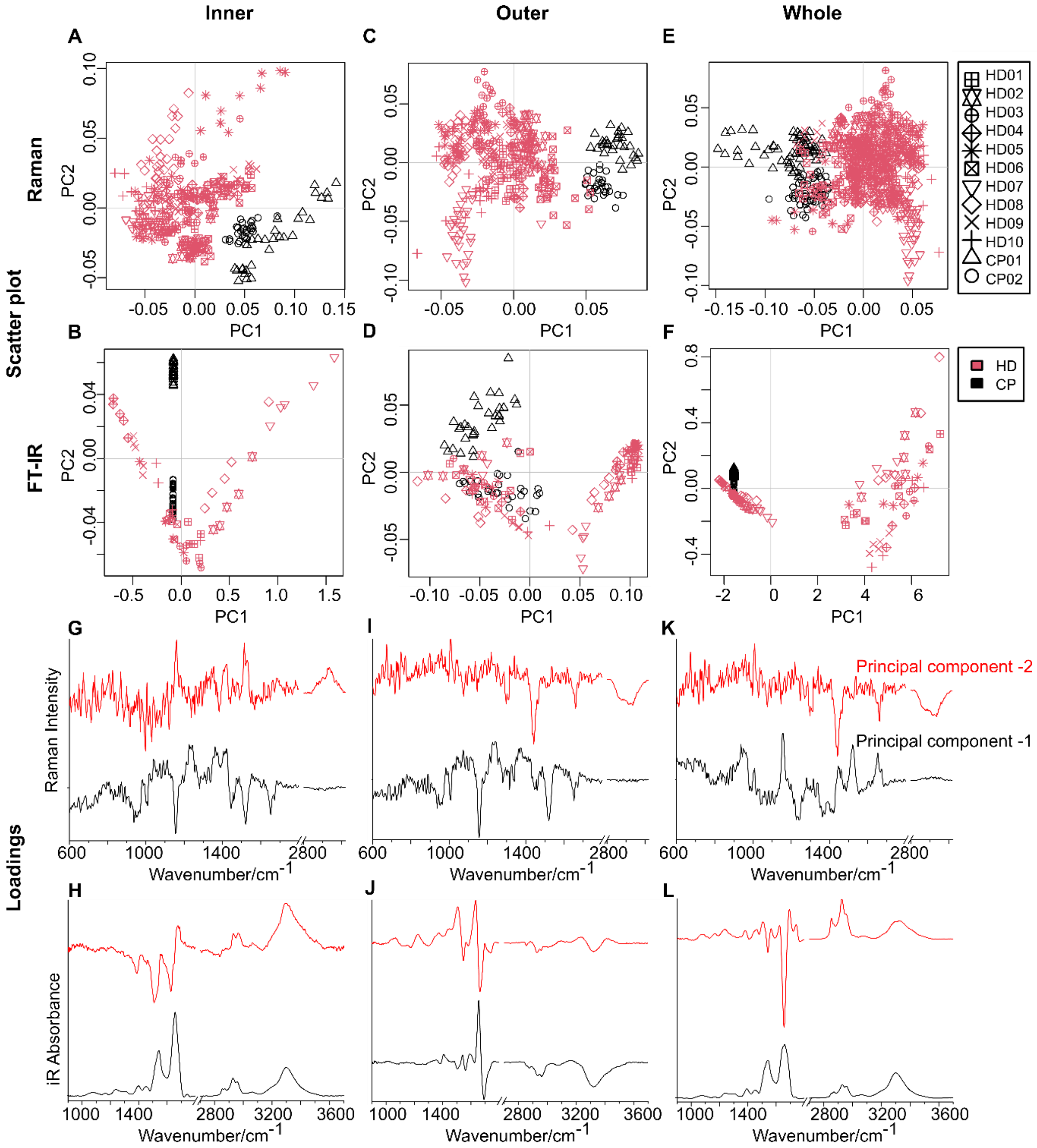

Figure S15. PCA grouping tendencies between healthy donors and cardiac patients calculated using vibrational spectroscopy data from dried serum sample droplet. PCA analysis of Raman (first row) and FT-IR spectra (second row) collected from (**A** and **B**) inner region and (**C** and **D**) outer regions from the dried serum sample droplet for nine different healthy donors (red color and individual donors are denoted by symbols) and two cardiac patients: CP01 is ICM patient and CP02 is HF patient (black color and individual cardiac patients are denoted by symbols). (**E** and **F**) combined analysis of the vibrational spectra collected from both inner and outer region. The principal components 1 (black) and 2 (red) for Raman (**G, I** and **K**) and FT-IR data (**H, J** and **L**).
